# My willpower belief and yours: Investigating dyadic associations between willpower beliefs, social support, and relationship satisfaction in couples

**DOI:** 10.1177/08902070231220416

**Published:** 2023-12-14

**Authors:** Zoë Francis, Rebekka Weidmann, Janina L. Bühler, Robert P. Burriss, Jenna Wünsche, Alexander Grob, Veronika Job

**Affiliations:** 1 Department of Psychology, 1011 University of the Fraser Valley, Abbotsford, BC, Canada; 2 Department of Psychology, 3078 Michigan State University, East Lansing, MI, USA; 3 9182 Johannes Gutenberg University Mainz, Mainz, Germany; 4 Department of Psychology, 27209 University of Basel, Basel, Switzerland; 5 40033 German Centre of Gerontology, Berlin, Germany; 6 27258 University of Vienna, Wien, Austria

**Keywords:** willpower beliefs, relationship satisfaction, social support, actor–partner interdependence model, implicit theories about willpower

## Abstract

A limited willpower belief describes the belief that one’s willpower is depletable and that mental exertion results in a diminished self-control capacity. Limited willpower beliefs have been associated with detrimental personal outcomes (such as poorer goal progress) and may even be related to a lower quality of one’s romantic relationship. With dyadic survey data from 745 couples across 14 days, we investigated how willpower beliefs of both partners were associated with their provision and receipt of social support, as well as their relationship satisfaction. We also examined whether partners with more similar willpower beliefs tended to have higher relationship satisfaction. A limited willpower belief was indeed associated with less provision of both instrumental and emotional support, according to both partners’ perspectives, and was also associated with a lower likelihood of receiving instrumental support. A limited willpower belief negatively correlated with one’s own relationship satisfaction, but partner effects were not significant. While couples’ willpower beliefs were more similar to each other than would be expected by chance, degree of similarity in willpower beliefs was not related to relationship satisfaction. Future research should examine the mechanisms via which willpower beliefs are involved in romantic relationships, potentially through impacting the exchange of support.

People perceive their self-control in different ways. Some report that their self-control is drained by strenuous mental effort, known as a belief in *limited willpower*. Others feel that their self-control is self-sustaining, known as a belief in *non-limited willpower* (Job et al., 2010; Mukhopadhyay & Johar, 2005). Willpower beliefs, which are typically measured on a continuum, are relatively stable individual differences (Bernecker et al., 2017), although they can also shift across the lifespan and depending on one’s circumstances (Job et al., 2018). Most often, willpower beliefs have been explored in relation to personal endeavours like goal-pursuit and perseverance. However, the ways that people experience and think about their willpower can also be consequential for (and may also be affected by) one’s close relationships (Francis & Job, 2018). Many behaviours that are important for building and maintaining interpersonal relationships can be effortful – from the provision of support (Gosnell & Gable, 2017), to self-sacrifice (Righetti & Impett, 2017), to planning shared experiences (Girme et al., 2014) – and these behaviours may be affected by people’s experiences of mental fatigue or depletion. Because people who believe that their willpower is limited tend to experience more mental fatigue (Francis et al., 2020, 2021), they may feel like they have less energy to use for building or maintaining their romantic relationships, potentially resulting in lower relationship satisfaction both for themselves and for their romantic partners. In the other causal direction, people in more supportive and satisfying relationships might feel more autonomy and self-efficacy, and feel that providing support to their partner (even when it is difficult) brings a sense of vitality. As a consequence, their willpower beliefs may grow to be more non-limited (Job et al., 2018).

Prior research has examined associations between willpower belief and *intentions* to provide support to one’s romantic partner (Francis et al., 2020; Francis & Job, 2021). However, neither actual support provision nor relationship satisfaction have been examined in relation to willpower beliefs. The present study uses dyadic data – where reports are provided from both members of a couple – to examine the associations between both partners’ willpower beliefs and the provision of social support. We further examine how couples’ willpower beliefs, including the similarity between the couple’s beliefs, might relate to relationship satisfaction. We use day-level data, collected each day for two weeks, to give insight on how willpower beliefs relate to people’s daily experiences of their romantic relationships.

## Willpower beliefs and support provision

One potential way that willpower beliefs may affect romantic relationships is by influencing how often individuals provide social support to their romantic partners. People who more strongly believe that their willpower is limited tend to experience more fatigue and negative emotion than those who believe their willpower is non-limited (Bernecker et al., 2017; Francis et al., 2021), and these negative affective states are associated with reduced provision of support (Iida et al., 2008). Two studies examining intentions to provide support to one’s romantic partner found results consistent with these associations; those with more limited willpower beliefs had weaker intentions to support their partner in response to hypothetical vignettes (Francis & Job, 2021) and when asked about their intentions to provide support in the upcoming hours (Francis et al., 2020). While intentions do not always correspond with actual behaviour (Gollwitzer & Sheeran, 2006; Sniehotta, 2009), these studies provide initial evidence that people with limited willpower beliefs may be less likely to provide support to their romantic partners, with potentially negative consequences for their relationships.

However, these initial studies on willpower beliefs and support provision were limited in three ways. First, both Francis et al. (2020) and Francis and Job (2021) measured intended or hypothetical support provision, rather than measuring actual provided support. Not only do intentions not always solidify into actions but willpower belief may even moderate the relationship between intentions and behaviour (Francis et al., 2021), making it especially difficult to make inferences about behaviour from data on intentions. The current research thus examines actual provided support, rather than merely intentions to support.

Second, previous research included only one person from a couple, and thus could speak neither to the experiences of the participants’ romantic partners nor to the role of the partner’s willpower belief. Relationship dynamics, including the exchange of social support and relationship satisfaction, arise from the characteristics of both partners as well as from the interactions between them (e.g., Iida et al., 2008; Levesque et al., 2014). Through analyzing dyadic data, we can explore how the willpower belief of each person is associated with the outcomes reported by both partners. The ways that one person’s willpower belief affects their own relationship satisfaction or reports of support are referred to as *actor effects*^1^ (Campbell & Stanton, 2015; Kenny et al., 2006). When one person’s willpower belief affects their partner’s relationship satisfaction or reports of support, these are referred to as *partner effects*. Prior research has only examined the relationship between support provision and one’s own willpower belief (i.e., actor effects). Partner effects of willpower beliefs have not been previously examined, but may be hypothesized to exist. For example, people may be more likely to provide support to their partner if their partner has a limited willpower belief and is experiencing fatigue. Or, considering the other direction of causality, if someone’s *partner* has low relationship satisfaction and efforts to improve the relationship are fruitless, one may start feeling like their energy is depleted and their own willpower belief may shift to become more limited.

The present study further differentiates between *instrumental support*, the provision of practical and concrete assistance, and *emotional support*, the provision of reassurance and warmth (Taylor, 2011). Prior work on willpower and social support has focused on emotional support (Francis & Job, 2021; Gosnell & Gable, 2017) or has measured support as an amalgamation of instrumental and emotional support behaviours (Francis et al., 2020), rather than examining each type of support separately, yet instrumental and emotional support are distinct behaviours that are associated with distinct personality traits and individual differences. For example, the provisions of instrumental and emotional support are associated with different facets of trait empathy (Devoldre et al., 2010). These two types of support may be unequally associated with willpower belief, depending on which type of support requires more effort to provide or which type of support contributes more to the formation of one’s willpower belief. We next theorize about the relative effortfulness of providing these two types of support.

### Effortfulness of instrumental versus emotional social support

Providing emotional support is often difficult. Empathizing is experienced as demanding (Cameron et al., 2019), feeling compassion is even more so (Scheffer et al., 2021), and providing emotional support requires focused attention (Collins et al., 2014). Attempting to reassure or comfort someone without sufficient effort or attention can be perceived as inauthentic or uncaring, and there is always the possibility of misinterpreting one’s partner’s emotions or desires, which can have negative repercussions for one’s relationship (Sened et al., 2017). Emotional support is typically provided during times when one’s partner is experiencing stress or other negative emotions; when these negative emotions are shared between partners, the support provider then may need to effortfully regulate their own emotions to effectively provide support (Cutrona & Russell, 2017). While people readily empathize and support close others, this is not necessarily because empathizing itself is easy but is because the rewards are greater (Ferguson et al., 2021). There is thus good reason to expect that people with limited willpower beliefs – who ration their expenditure of effort – may provide less emotional support than those with non-limited willpower beliefs.

Providing *instrumental* support may require varying amounts of physical or mental effort or may be relatively effortless, depending on the specific task conducted – forms of instrumental support vary widely (Egan, 2020). Some instrumental support tasks, like making a stressful phone-call on behalf of one’s partner, may require similar degrees of emotion-regulation and mental effort as emotional support. But other instrumental support tasks, like ordering take-out for one’s spouse or doing extra household chores, may require relatively little mental or physical effort. Additionally, some forms of instrumental support may be relatively routine or habitual, further reducing the effort required. In prior research, participants with a limited willpower belief *initially* struggled more to regularly complete instrumental self-care tasks, compared to participants with a more non-limited belief (Bernecker & Job, 2015). However, with time and experience, these instrumental tasks became more familiar and easier, and willpower belief was no longer associated with completion of self-care tasks. A similar process may occur with instrumental support – if people frequently support their romantic partner with the same types of instrumental tasks (e.g. automatically cooking meals for one’s partner on days when their partner is overwhelmed), they may be able to do these instrumental tasks with relatively little effort. Willpower belief thus may not be associated with instrumental social support, or at least not as much as emotional support. On the other hand, some instrumental support tasks do require effort, making the ‘typical’ effortfulness of instrumental support unclear and inconsistent. Of note, however, habitual instrumental support tasks may be less likely to be noticed or reported by either partner – if only non-habitual, effortful forms of instrumental support are noticed and reported, then we may still see a negative association between limited willpower belief and the provision of instrumental support.

## Willpower belief and relationship satisfaction

**Figure 1.**
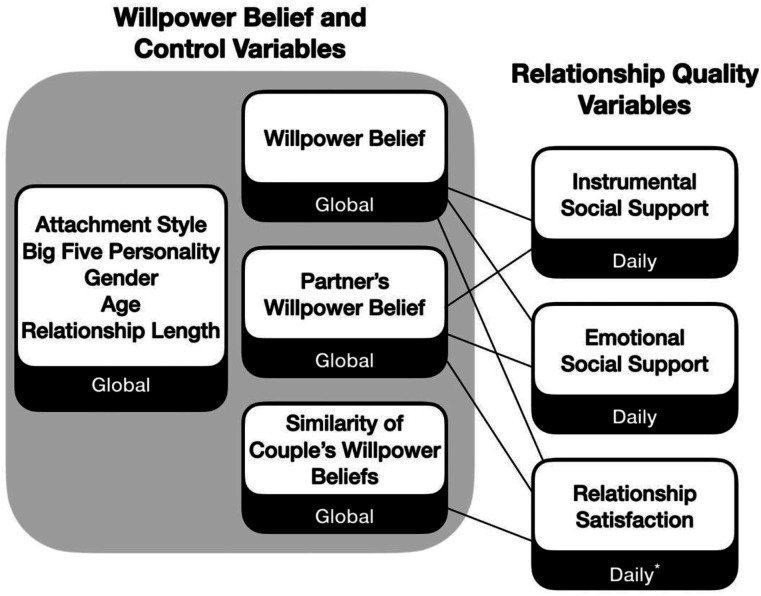
Overview of variables. Note. *Relationship satisfaction was also measured using a global measure on the final day of the two-week wave. Results are consistent with the daily measure of relationship satisfaction; see Supplemental Materials for analyses.

Beyond examining associations between willpower belief and support, we hypothesize that a more limited willpower belief may be associated with lower relationship satisfaction (Figure 1). If people with more limited willpower beliefs are less likely to provide support or engage in other effortful relationship maintenance behaviours, these beliefs may be related to poorer relationship quality overall (Brunstein et al., 1996; Feeney & Collins, 2015). Willpower belief may be associated with relationship satisfaction for both oneself (i.e. actor effect) and one’s partner (i.e. partner effect). Due to the lack of prior dyadic research on willpower belief, only the actor effect has been previously explored; Francis and Job (2018) found a small non-significant negative correlation (r = −.09) between limited willpower belief and relationship satisfaction. If the partners of those with limited beliefs receive less support from their partners, they may also report lower relationship satisfaction.

Willpower belief may also be related to relationship satisfaction through other mechanisms, beyond the exchange of social support. A limited willpower belief is typically associated with poorer life satisfaction and lower well-being (Bernecker et al., 2017). Because life satisfaction and relationship satisfaction are often strongly correlated (Gustavson et al., 2016; Molero et al., 2017), willpower belief may be especially likely to correlate with lower relationship satisfaction as an actor effect. Furthermore, when one member of a relationship experiences chronic negative affect, both partners can experience detrimental effects on their relationship satisfaction (e.g. in the case of depressive symptoms, Morgan et al., 2018). As limited willpower beliefs are associated with more negative affect (Francis et al., 2020, 2021), both those who hold these beliefs and their romantic partners may have poorer relationship satisfaction. Thus, regardless of the associations between support provision and willpower belief, we aim to investigate both the actor and partner effects of willpower belief on relationship satisfaction.

## Similarity hypotheses

A separate possibility is that couples with more similar willpower beliefs will have higher relationship satisfaction. Similarity or congruency effects on relationship satisfaction are seen for some, but certainly not all, individual differences. For example, similarity in life goals and values positively (but weakly) predict relationship satisfaction (Arránz Becker, 2013), as does similarity in political orientation (Leikas et al., 2018) and similarity in chronotype (i.e. trait morningness-eveningness; Jocz et al., 2018). However, similarity in the Big Five personality traits typically does not predict relationship satisfaction (Leikas et al., 2018; Weidmann et al., 2017), nor does similarity in trait self-control (Vohs et al., 2011). Similarity in willpower belief may be relevant to relationship satisfaction (like similarity in values) or it may be largely irrelevant (like similarity in trait self-control).

If similarity in willpower belief is associated with higher relationship satisfaction, the association could be supported by at least two possible mechanisms. First, it is possible that couples with similar beliefs have more aligned preferences for what activities and goals to engage in, leading to less potential for conflict and higher relationship satisfaction. People do not only hold personal goals but also hold goals for their partners and for their relationship (Fitzsimons et al., 2015), and shared perceptions of goal progress positively predicts relationship satisfaction (Avivi et al., 2009). If partners with more similar willpower beliefs have more congruent and compatible goals, they may then have higher relationship satisfaction. For example, two people with similarly limited willpower beliefs might both agree that trying to eat more healthily is an appropriate goal, and each believe that the pursuit of that goal will unfortunately deplete their willpower. With a common understanding that willpower is limited, they may both be more understanding of one another’s goal lapses and would not expect one another to pursue additional goals or life changes while simultaneously eating healthily. If, instead, one person in the couple has a non-limited willpower belief (resulting in mis-matched beliefs), they might think that pursuing the goal of eating healthier is an excellent opportunity to also start exercising and reduce alcohol consumption, to take advantage of the shifting lifestyle to pursue multiple simultaneous goals. Differing perceptions of how to set, achieve, and pursue goals might lead to conflict and even lower relationship satisfaction.

Alternatively, because willpower beliefs change across time (Sieber et al., 2019), someone’s beliefs about willpower may begin to more closely resemble their partner’s as their relationship progresses, they spend time together, and have more shared goals. This convergence could be stronger for those with higher relationship satisfaction and for those who have been in their relationship for longer. In this account, relationship satisfaction (and relationship duration) results in more similar willpower beliefs. However, convergence effects on other beliefs, attitudes, and traits are typically small or non-existent (see Luo, 2017 for review). Because similarity in willpower belief across dyads has not previously been examined, based on inference from prior literature, similarity may either be positively related to relationship satisfaction or may be unrelated. Nevertheless, data exploration was driven by the working hypothesis of a similarity effect.

## Study hypotheses

In the following study, we use day-level dyadic data from a large longitudinal study to investigate the following hypotheses.

Hypothesis 1 A more limited willpower belief will be associated with less provision of emotional support, according to both one partner’s report of provided support and according to the other partner’s report of received support.

Hypothesis 2 A more limited willpower belief may be associated with less provision of instrumental support, according to both one partner’s report of provided support and according to the other partner’s report of received support. The association between willpower belief and instrumental support may be weaker than the association between willpower belief and emotional support (Hypothesis 1).

Hypothesis 3 A more limited willpower belief will be associated with lower relationship satisfaction for both oneself and one’s partner.

Hypothesis 4 Couples with more similar willpower beliefs will have higher relationship satisfaction (due to a similarity effect).

### Related variables (covariates)

Because willpower belief is individual difference that correlates with other traits (Jędrzejczyk & Zajenkowski, 2020), we conducted additional analyses to examine whether willpower belief has unique associations with social support and relationship satisfaction, beyond potentially correlated individual difference variables. Following prior work (Francis et al., 2020), we included both the Big Five personality traits and attachment style as covariates. A belief in limited willpower is moderately correlated with neuroticism (*r* = .27) and may also negatively correlate with extraversion and openness (Francis et al., 2020; Jędrzejczyk & Zajenkowski, 2020). These personality traits are themselves be related to relationship outcomes (Malouff et al., 2010; Weidmann et al., 2017). Anxious and avoidant attachment styles were likewise included as covariates, as attachment style is a consistent predictor of both perceived support provision (Moreira et al., 2003) and relationship quality (Candel & Turliuc, 2019; Li & Chan, 2012).

Demographic variables – especially relationship length, age, and gender – may also be relevant to understanding of support provision and relationship satisfaction. For example, as described above (see ‘Similarity Hypothesis’, above), partners’ willpower beliefs might converge across time, correlating more strongly as relationship length (and age) increases. Willpower beliefs also change across the lifespan, with older adults holding more nonlimited beliefs in willpower (Job et al., 2018). We thus include age and relationship length as relevant control variables.

Lastly, gender and corresponding gender norms can also influence the provision of support; women and men may be objectively similarly effective at providing support (Neff & Karney, 2005; Sullivan et al., 2010; Verhofstadt et al., 2007), although women may provide more emotional and instrumental support according to self-report (Verhofstadt et al., 2007). Additionally, women may provide more support during times of stress (Bodenmann et al., 2015) and better respond to changing needs (Neff & Karney, 2005). We thus also test for potential gender effects and include gender as a covariate in our models.

## Methods

### Participants

This study used questionnaire and daily responses from the second wave of an online four-wave longitudinal study of couples, *Processes in Romantic Relationships and Their Impact on Relationship and Personal Outcomes* (CouPers)^2^. The current investigation makes use of only the second wave of data for this larger study because the second wave included the measure of willpower belief. German-speaking romantic couples, who had been in a relationship for at least one month, were recruited from the University of Basel, local communities, and through Facebook advertisements targeted to users in Switzerland, Austria, and Germany (full recruitment information in Bühler et al., 2020). Sixty percent of participants were residents of Germany, 27% of Switzerland, 11% of Austria, .2% resided in other countries, and 1% did not indicate.

The dataset included 1490 participants (745 female–male couples), who each completed up to 14 days of daily surveys. On average, participants in this sample completed 72% of surveys for the current wave (28% missingness). At wave 1, participants were an average age of 32.71 years old (SD = 14.00, median = 27.00) and ranged from 18 to 81 years. They had been in their current relationships for an average of 8.71 years (SD = 10.66, median = 4.75), with a range from 2 months to 52 years. Twenty-nine percent of participants had at least one child. Participants were employed full-time (42%), in school (28%), employed part-time (20%), out of the labour market (8%), or unemployed (2%). They also reported a variety of personal incomes: no personal income (13%), under 20,000 (35%), from 20,000 to 40,000 (22%), from 41,000 to 60,000 (10%), 61,000 to 80,000 (8%), 81,000 to 100,000 (3%), above 100,000 (4%), or unknown/not provided (6%). The willpower belief measure was completed by 1331 of the participants.

### Preregistration and power analysis

While this study was not publicly preregistered, hypotheses were recorded as part of the request for data usage, prior to accessing the dataset. These *a-priori* hypotheses and the commented R analysis code are both available at osf.io/c2z9y/. We report all exclusions.

The sample size of the dataset was originally selected to be sufficiently powered to investigate associations between personality and relationship outcomes and to be able to conduct more elaborate statistical models to examine change across time. In regards to the current hypotheses, sensitivity analyses suggest that the analyzed sample size (636 couples with complete data) had 80% power to detect small actor effects and partner effects of at least b = .08 for analyses of global relationship satisfaction using the APIM (Ackerman & Kenny, 2016). Additionally, we had 80% power to detect within-couple correlations of r = .11 (calculated with G*Power; Faul et al., 2009).

### Procedure

Participants had previously participated in the first wave of the study, four to six months before the second wave of data that is used in the present analyses. Participants provided informed consent prior to participating in the first wave of data collection. The attrition from the first wave to the second wave was 24% of individuals (26% of couples).

On the first day of the second wave, participants were asked to complete a battery of questionnaires. They then received short daily surveys each day for two weeks; these daily surveys were sent via email at 4pm each day and took an average of 6 minutes to complete. After 14 days of daily measures, they received a second set of questionnaires. All measures and the full procedure of the study are available at osf.io/59vhs.

All surveys were completed online. Participants were compensated with gift cards after each wave (of 20 CHF/€) and could also choose to receive personal feedback on one of the survey measures selected by the researchers. Ethical approval for the study was received from the Institutional Review Board of the Department of Psychology at the University of Basel.

### Individual difference measures

#### Willpower beliefs

Willpower belief was measured on the final day of the two-week wave, using the 12-item willpower belief questionnaire (Job et al., 2010; scale validation and lack of measurement invariance described in Napolitano & Job, 2018); we further discuss the timing of this measure in the Discussion limitations section. Each item was measured on a six-point scale ranging from (1) *strongly agree to* (6) *strongly disagree*. Responses were recoded so that higher scores indicated a more limited willpower belief, and then averaged. The highest-loading questions were ‘After a strenuous mental activity, you feel energized for further challenging activities’ and ‘After a strenuous mental activity, your energy is depleted and you must rest to get it refuelled again’ (reverse-coded).

The willpower belief questionnaire has two subscales that measure people’s beliefs about strenuous mental activity and about resisting temptations. Because we had no reason to believe that one specific subscale would be associated with support provision or relationship satisfaction more than the other, we collapsed across subscales (Di Maio et al., 2020; Job et al., 2018). The 12-item overall willpower belief measure had acceptable internal reliability (e.g. ω = .82 for women; ω = .76 for men; [McNeish, 2018; Sakaluk et al., 2021]), but the model fit for a single factor scale was not adequate and a two-factor model significantly improved the fit. Thus, while we primarily report on the analyses using the entire 12-item scale, separate analyses for each subscale are available in the Supplemental Materials. Factor loadings and structural models (for both one-factor and two-factor models) are also available in the Supplemental Materials. Note that, at the level of loading invariance, there was no significant measurement invariance across gender for the 12 items (*χ*^2^ = 19.16, *p* = .06) and the effect sizes for noninvariance for each item were small (d_MACS_ < .25; Sakaluk et al., 2021).

#### Control variables

To examine whether willpower belief is uniquely associated with social support and relationship satisfaction, personality traits, attachment style, age, gender, and relationship length were also measured and included in the current analyses as control variables.

##### Big five personality traits

Personality traits were measured on the first day of the wave via the German translation of the Big Five Personality Inventory (John & Srivastava, 1999; Rammstedt & Danner, 2016). Each trait was measured with eight to ten items, with responses recorded from (1) *does not apply at all to* (5) *applies completely and averaged*. Each trait had good reliability (omegas for women/men; openness ω = .84/.83; conscientiousness ω = .86/.85; extraversion ω = .88/.88; agreeableness ω = .77/.78; neuroticism ω = .87/.85).

##### Attachment style

Attachment style was measured on the first day of the wave, using the Experiences in Close Relationships–Relationship Structures questionnaire (ECR–RS; Fraley et al., 2011). This questionnaire measures two dimensions of attachment style: anxious attachment (three items, ω = .77 for women/.77 for men) and avoidant attachment (six items, ω = .84/.84). Highly loading items include, ‘I often worry that my partner doesn’t really care for me’, measuring anxious attachment, and ‘I talk things over with my partner’ (reverse-scored), measuring avoidant attachment. Responses were measured on seven-point Likert scales, from (1) *completely inapplicable to* (7) *completely applicable*, and averaged for each of the two dimensions. The ECR–RS has good measurement invariance across gender and age (Gray & Dunlop, 2019).

##### Demographics

The demographic measures – including age, relationship length, and gender – were measured in the first wave of the study, 4–6 months prior to the daily surveys analyzed here. These variables are further described above, under Participants.

### Daily relationship quality measures

#### Daily social support

Each day, participants reported both their received and *provided* emotional and instrumental (practical) support (Shrout et al., 2006). Participants were given the following instruction: ‘Please indicate any help you received with a worry, problem, or difficulty from your partner today. Help can be emotional (e.g. listening and comforting) or practical (e.g. doing something concrete)’. Their received emotional and instrumental support were measured one item each (i.e. ‘My partner provided practical [emotional] support to me today’), and responses were binary (yes = 1, no = 0). They then responded to two questions, which asked whether they provided emotional or instrumental support to their partner that day, again with the same binary response options.

#### Daily relationship satisfaction

Relationship satisfaction was measured on the daily level using a single question: ‘Overall, how satisfied were you today with your relationship with your partner?’. Participants selected one of five response options, which varied from (1) *very dissatisfied to* (5) *very satisfied*.

### Analysis

Analyses of the daily survey data were conducted with two-level hierarchical models, with daily surveys nested within couple (Iida et al., 2010; Nikitin et al., 2021). We included random intercepts for each couple. Because measures of social support were binary, we used the glmer function from lme4 version 1.1-27.1 (Bates et al., 2015), specifying a binomial distribution, when conducting models predicting either provided or received social support. If a model did not converge with default optimizers, we specified a bobyqa optimizer (model-by-model information available in the R analysis code; osf.io/54z8m). To predict measures of daily relationship satisfaction, we used the lmer function, also from lme4. The lmerTest package version 3.1-3 was used to estimate degrees of freedom and *p*-values (Kuznetsova et al., 2017).

We first report the results of models with the outcome – relationship satisfaction or support – predicted only by the two predictors of willpower belief held by oneself (actor effect) and the willpower belief held by one’s partner (partner effect). We then report the results of larger models that included willpower belief along with demographic variables, personality traits, attachment style variables of both oneself and one’s partner (a set of twenty simultaneous predictors) to investigate whether willpower belief has unique shared variance with these daily relationship outcomes.

We tested for similarity effects both using dyadic response surface analyses (analyses and results described in the Supplemental Materials) as well as by calculating the absolute difference in willpower beliefs between both members of a couple and using the difference score as a predictor of relationship satisfaction (results below). Results were consistent across both sets of analyses.

All analyses were conducted using R version 4.0.4 (R Core Team, 2021).

## Results

### Provision and receipt of social support

**Figure 2.**
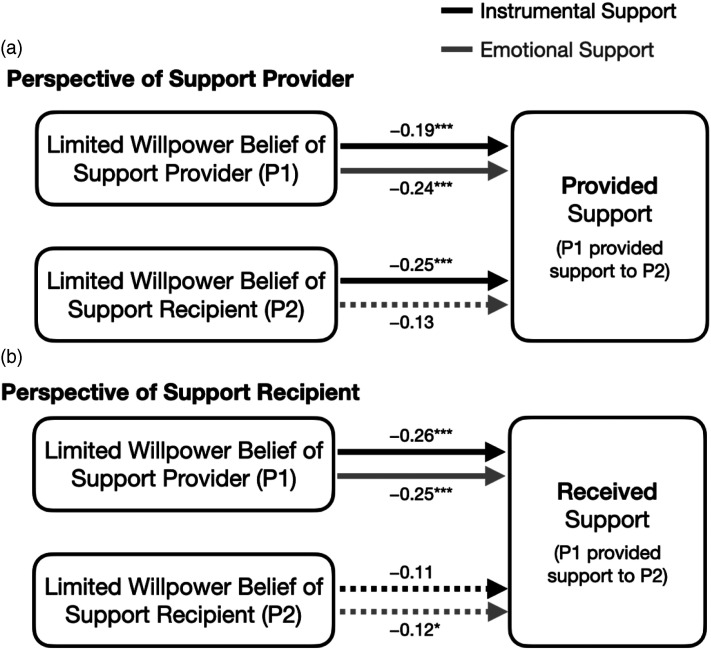
Summary of four statistical models examining how support was predicted by the willpower beliefs of both relationship partners.Note. Black lines show the effects for instrumental support and grey lines show effects for emotional support. Dashed lines are not statistically significant at *p* < .01. ***p < .001; **p < .01; *p < .05. See Supplemental Materials for these associations by willpower belief subscales (Strenuous Mental Activity and Resisting Temptation).

We first examined whether those with a more limited willpower belief were less likely to provide support to their romantic partners. Provided emotional support was predicted by the provider’s own willpower belief, with those with more limited willpower beliefs being less likely to report having provided emotional support (Figure 2; actor effect, *b* = −.24, SE = .07, *z* = −3.70, *p* < .001). Critically, this was validated by their partner’s reports of received emotional support; people were less likely to report having received emotional support if their partner had a more limited willpower belief (partner effect; *b* = −.25, SE = .06, *z* = −4.31, *p* < .001).

Instrumental support was likewise predicted by the provider’s willpower belief, both according to reports of the provider (actor effect; *b* = −.19, SE = .06, *z* = −2.95, *p* = .003) and according to reports from the recipient (partner effect; *b* = −.26, SE = .06, *z* = −4.40, *p* < .001). These associations with instrumental support are not significantly different in magnitude than the above associations with emotional support (as the estimates differ by less than one standard error). Thus, extending from previous research, people who believe that their willpower is more limited were less likely to provide both emotional and instrumental support to their romantic partners. This was true from the perspectives of both partners in the relationship.

Does the willpower belief of the support *recipient* also matter? Both instrumental and emotional support were associated with the willpower belief of the support recipient in some analyses, but less strongly and less consistently. Emotional support was *less* likely to be provided to a recipient with a limited willpower belief, but the association only reached statistical significance when considering reports from the recipient (Figure 2; according to the provider, *b* = −.13, SE = .07, *z* = −1.90, *p* = .057, partner effect; according to the recipient, *b* = −.12, SE = .06, *z* = −2.09, *p* = .037, actor effect). Instrumental support was also less likely to be provided to a recipient with a limited belief, and the association only reached statistical significance when using reports from the provider (according to provider, *b* = −.25, SE = .06, *z* = −3.94, *p* < .001, partner effect; according to recipient, *b* = −.11, SE = .06, *z* = −1.83, *p* = .067, actor effect).

#### Additional trait predictors

We next examined whether willpower belief was a unique predictor of social support, above and beyond any effects of attachment style and personality (zero-order correlations available in Table 1), as well as demographic variables including relationship length, age, and gender. All these variables (see Table 2) were entered as simultaneous predictors, along with the partner’s report of received or provided support.

**Table 1.** Correlation Matrix for all Individual Difference Predictors.

			Women									Men								
		Mean (SD)	WP	N	E	C	O	Agr	Anx	Avo	RS	WP	N	E	C	O	Agr	Anx	Avo	RS
Women	Willpower	3.38 (.7)																		
	Neuroticism	2.81 (.8)	.29																	
	Extraversion	3.52 (.75)	−.17	−.33																
	Conscientious	3.64 (.65)	−.37	−.31	.22															
	Openness	3.58 (.65)	−.14	−.13	.27	.02														
	Agreeableness	3.67 (.55)	−.10	−.36	.18	.20	.13													
	Anx. Attach	2.26 (1.25)	.17	.33	−.24	−.15	−.08	−.23												
	Avoid. Attach	2.12 (.95)	.04	.10	−.14	−.09	−.04	−.14	.36											
	Rel. Sat	4.28 (.59)	−.03	−.13	.12	.09	−.01	.14	−.39	−.56										
Men	Willpower	3.38 (.7)	.08	.04	−.11	−.03	−.01	−.05	.02	.00	−.07									
	Neuroticism	2.81 (.8)	.00	−.09	−.03	−.08	.03	−.08	.03	.12	−.19	.24								
	Extraversion	3.52 (.75)	−.06	−.06	.02	.07	−.04	.06	−.04	−.06	.12	−.17	−.28							
	Conscientious	3.64 (.65)	−.09	−.20	.15	.10	.02	.19	−.09	−.06	.06	−.26	−.27	.18						
	Openness	3.58 (.65)	−.04	−.04	.10	−.02	.12	.06	−.05	−.05	.08	−.23	−.11	.31	.04					
	Agreeableness	3.67 (.55)	−.04	−.09	.04	.10	.04	.08	−.13	−.09	.12	−.01	−.34	.13	.16	.05				
	Anx. Attach	2.26 (1.25)	.01	.08	−.08	−.07	−.01	−.13	.20	.28	−.35	.07	.31	−.13	−.12	−.06	−.25			
	Avoid. Attach	2.12 (.95)	.04	.14	−.08	−.06	−.04	−.15	.25	.35	−.39	.07	.18	−.23	−.10	−.08	−.24	.41		
	Rel. Sat	4.28 (.59)	−.04	−.15	.09	.07	−.02	.15	−.30	−.32	.57	−.13	−.24	.11	.10	.12	.19	−.36	−.52	
	Rel. Length		−.14	−.12	.08	.19	−.01	.14	−.07	.01	−.08	−.14	−.13	.07	.19	−.01	.13	−.07	.004	−.07

Note. Correlations ≥ |.08| are significant at *p* < .05 and correlations ≥ |.12| are significant at *p* < .001. Willpower = Limited Willpower Belief.

**Table 2.** Models Predicting Support and Relationship Satisfaction With Simultaneous Predictors.

	Model 1. Provided Instrumental Support b (SE)	Model 2. Provided Emotional Support b (SE)	Model 3. Received Instrumental Support b (SE)	Model 4. Received Emotional Support b (SE)	Model 5. Daily Relationship Satisfaction b (SE)
Actor effects					
Limited willpower	.02 (.08)	.0004 (.08)	−.01 (.07)	−.03 (.07)	−.05 (.02)*
Neuroticism	.14 (.09)	.04 (.09)	.13 (.08)	.29 (.08)***	−.05 (.02)*
Extraversion	.15 (.08)	.25 (.08)**	.11 (.07)	.23 (.07)***	.002 (.02)
Conscientiousness	−.01 (.09)	−.05 (.09)	−.10 (.09)	−.10 (.08)	.001 (.02)
Openness	.22 (.08)**	.37 (.08)***	.15 (.08)	.23 (.08)**	.02 (.02)
Agreeableness	.30 (.11)**	.13 (.10)	.36 (.10)***	.25 (.09)**	.05 (.03)
Anxious attach	.09 (.05)	.07 (.05)	−.01 (.04)	.05 (.04)	−.04 (.01)***
Avoidant attach	−.16 (.06)**	−.23 (.06)***	−.17 (.06)**	−.12 (.06)*	−.18 (.02)***
Age	.01 (.01)	.03 (.01)***	.01 (.01)*	.01 (.01)	.001 (0)
Gender	−.05 (.08)	.19 (.09)*	.74 (.08)***	.59 (.09)***	.0002 (.02)
Relationship length	.03 (.01)**	.001 (.01)	.02 (.01)*	.01 (.01)	.001 (0)
Partner effects					
Limited willpower	−.13 (.08)	−.03 (.08)	−.09 (.07)	−.17 (.07)*	.01 (.02)
Neuroticism	.16 (.09)	.32 (.09)***	.02 (.08)	.03 (.08)	−.02 (.02)
Extraversion	−.03 (.08)	.002 (.08)	−.01 (.07)	.06 (.07)	−.03 (.02)
Conscientiousness	.02 (.09)	.06 (.09)	−.04 (.09)	−.05 (.08)	−.01 (.02)
Openness	.13 (.08)	.14 (.08)	.06 (.08)	.12 (.07)	.03 (.02)
Agreeableness	.29 (.11)**	.17 (.1)	.05 (.1)	.18 (.09)*	−.03 (.03)
Anxious attach	−.01 (.05)	.01 (.05)	−.02 (.05)	−.08 (.04)	−.05 (.01)***
Avoidant attach	−.04 (.06)	−.14 (.06)*	−.04 (.06)	−.14 (.06)	−.05 (.02)**
Partner’s DV report^+^	.16 (.08)	.34 (.09)***	.17 (.07)*	.57 (.09)***	.14 (.01)
ICC	.30	.24	.28	.18	.12

Note. Unstandardized coefficients and standard errors from multi-level models with random intercepts for each couple. Models 1 through 4 specified a binomial distribution, and ICCs these models were estimated from the random intercept logistic model (Wu et al., 2012). Bolded values are significant at α = .05. *p < .05; **p < .01; ***p < .001^+^ the partner’s report of provided support, received support, or daily relationship satisfaction was also included as a covariate (e.g., partner’s report of provided instrumental support was included in Model 1, partner’s report of relationship satisfaction was included in Model 5).

First, we consider the perspective of the support provider (Table 2, Models 1 and 2). After considering other individual variables, one’s own willpower belief did not significantly predict how often one reported providing either emotional or instrumental support. Instead, reports of provided instrumental and emotional supports were predicted by the provider being more open to experience and less avoidantly attached (Table 2, Models 1 and 2). Provided emotional support was further predicted by extraversion, while provided instrumental support was further predicted by agreeableness. Women were more likely to provide emotional support, and older age (or relationship length) were also predictive of providing more support.

We next describe the results from the perspective of the support recipient. Again, one’s own willpower belief was no longer a significant predictor after including the covariates. However, people were less likely to report receiving support if their partner (i.e. the support provider) had a more limited willpower belief (*b* = .17, SE = .07, *z* = 2.46, *p* = .013). Received emotional support was simultaneously predicted by one’s partner being higher in agreeableness and lower in attachment avoidance. People were also more likely to report having received emotional support if they themselves had higher neuroticism, higher extraversion, higher openness, higher agreeableness, less avoidant attachment, and were female (actor effects, Table 2 Model 4).

With all predictors entered simultaneously, none of one’s partners’ traits significantly predicted receiving instrumental support. The recipient’s own levels of agreeableness, age, gender, and relationship length and avoidant attachment all predicted the likelihood of reporting having received instrumental support (actor effects, Table 2, Model 3).

Finally, we had earlier found that the willpower belief of the support recipient predicted instrumental support, as reported from the perspective of the support provider. After controlling for other individual differences, this relationship was no longer significant. Instead, participants were more likely to report that they provided instrumental support to their partner if their partner was higher in agreeableness, and more likely to provide emotional support if their partner was higher in neuroticism and lower in avoidant attachment (Table 2, Model 1 and 2 partner effects).

Thus, while willpower belief was a significant predictor of support provision as a single predictor (see above) in all four statistical models, much of its predictive value was shared across other trait measures and individual differences. Willpower belief may be a more useful predictor when considering the perspective of the support recipient (as a partner effect), but a less unique predictor when considering the perspective of the support provider (an actor effect).

### Relationship satisfaction

Is a limited willpower belief also related to poorer relationship satisfaction, for either oneself or one’s partner? We investigated the possible association between willpower belief and relationship satisfaction at the daily level (see Supplemental Materials for analysis of relationship satisfaction at the global level; results are consistent with the presented day-level analyses).

Daily relationship satisfaction was significantly predicted by one’s own willpower belief (actor effect; *b* = −.08, SE = .02, *t*(783) = −4.20, *p* < .001), with a more limited willpower belief correlating with lower relationship satisfaction. There was no significant partner effect of willpower belief on relationship satisfaction (*b* = −.01, SE = .02, *t*(785) = −.77, *p* = .44). As expected, the relationship satisfaction of one member of the couple was also predicted by the relationship satisfaction of the other (*b* = .12, SE = .009, *t*(11610) = 13.39, *p* < .001, included in model as covariate)^3^.

#### Additional trait predictors

We next tested whether the above actor effect of willpower belief predicted daily relationship satisfaction above and beyond the effects of the Big Five personality traits, attachment style, and the demographic variables. In this full model, a more limited willpower belief was still associated with poorer daily relationship satisfaction (*b* = .14, SE = .01, *t*(9172) = 13.06, *p* < .001). Other significant actor effects of personality on daily relationship satisfaction included neuroticism (*b* = −.05, SE = .02, *t*(548) = −2.22, *p* = .027), anxious attachment style (*b* = −.04, SE = .01, *t*(748) = −3.68, *p* < .001), and avoidant attachment style (*b* = −.18, SE = .02, *t*(894) = −11.61, *p* < .001).

The only significant partner effects on daily relationship satisfaction were from anxious attachment (*b* = −.05, SE = .01, *t*(759) = −4.11, *p* < .001) and avoidant attachment (*b* = −.05, SE = .01, *t*(886) = −3.03, *p* < .001). All actor and partner effects are presented in Table 2, Model 5.

### Similarity effects

Finally, we investigated whether couples who had more similar willpower beliefs to one another tended to report higher relationship satisfaction. The absolute value of the difference between willpower belief did not predict daily relationship satisfaction (*b* = −.001, SE = .03, *t*(638) = −.02, *p* = .99).^4^ Additional analyses using Response Surface Analysis (see Supplemental Materials) likewise found no evidence for similarity effects.

On average, people’s willpower beliefs were more similar to the willpower beliefs of their partners than would be expected by chance, but the correlation was small (*r* = .08, *t*(634) = 2.10, *p* = .035). Similarity in willpower belief was not significantly moderated by relationship length (*b* = −.003, SE = .003, *t*(598) = −.93, *p* = .35), failing to find evidence for the hypothesis that people’s willpower beliefs converge over time.

## Discussion

Beliefs about one’s willpower as more limited or non-limited have been rarely examined in the context of romantic relationships, despite willpower and self-regulation being implicated in the functioning and maintenance of interpersonal relationships (de Ridder et al., 2012; Finkel et al., 2016; Righetti & Finkenauer, 2011). With day-level data provided by a large sample of couples, we investigated how willpower belief was associated with the relationship outcomes of support provision and relationship satisfaction, as well as exploring the role of similarity in willpower beliefs. While similarity of a couple’s willpower beliefs did not correlate with either relationship measure, willpower belief itself was related to the provision of both emotional and instrumental support, the receipt of instrumental support, and one’s own relationship satisfaction.

People with more limited willpower beliefs provided support less frequently to their romantic partners, in line with earlier work on intentions to provide support (Francis et al., 2020; Francis & Job, 2021). Interestingly, a more limited willpower belief was associated with providing both less emotional support and less instrumental support, to similar extents. We hypothesized that emotional support may be more effortful than instrumental support and thus might be more strongly associated with willpower belief; however, this was not the case. Potentially, if an act of social support was sufficiently noteworthy and non-habitual for a participant to recall during the daily survey, it was likely to be at least somewhat effortful for the provider. These associations between willpower belief and support were not only found from the perspective of the support *provider* but were also confirmed by the support *recipient* (Figure 2). Although the partners of those with limited willpower beliefs received support less frequently, they did not experience poorer relationship satisfaction. Unexpectedly, people with more limited willpower beliefs were also less likely to receive instrumental support from their romantic partners. While we had not formally hypothesized associations with the willpower beliefs of the support recipient, we informally predicted that people with limited willpower beliefs would receive more support because they are more likely to experience fatigue and negative emotion (Bernecker et al., 2017; Francis et al., 2021) and people are more likely to receive social support on days when their mood is lower (Iida et al., 2008). Why then were people with limited beliefs less likely to receive instrumental support? Potentially, those who believe that their willpower is limited might be, on average, less busy and less likely to be engaging in demanding or difficult activities when around their partner, and thus less likely to require or receive instrumental support. People with limited beliefs are less likely to be physically active and more likely to rest and recover – particularly in the evenings and after experiencing demands (Francis et al., 2021; Job et al., 2015; Konze et al., 2018) – so perhaps their partners have fewer opportunities to assist them in concrete ways.

Alternatively, it could be that a lack of support from one’s partner *leads* to the development of a more limited willpower belief. Experimental studies have found that people who experience more effortful scenarios are more likely to report that their willpower is limited afterwards (Klinger et al., 2018), and willpower theories can shift across time, becoming more non-limited when people experience more autonomy (Job et al., 2018). Potentially, people who receive less support from their partners may experience higher demands or might experience their demands as more burdensome, thus experiencing more fatigue, and more readily believe that their willpower is limited. Future research should explore the potential causal relationships between willpower beliefs and the receipt of social support via longitudinal or experimental studies.

There were small but consistent associations between one’s own willpower belief and relationship satisfaction; people with a limited willpower belief tended to report poorer relationship satisfaction in their daily surveys (and had slightly poorer assessments of their overall relationship quality, as described in Supplemental Materials). This association between relationship satisfaction and willpower belief is similar in magnitude to the correlation reported in Francis et al. (2020), but the much larger sample size of the current study provides confidence that this association is indeed negative and small (95% CI of *r* = [−.02, −.12]). The association between willpower belief and lower relationship satisfaction may be due to those with limited beliefs experiencing more negative affect and lower well-being overall (Bernecker et al., 2017), which can colour their experience and perceptions of their relationship. Indeed, an exploratory mediation model (available in the Supplemental Materials) found that participants with more non-limited willpower beliefs reported lower positive affect and higher negative affect, and these self-reported affect measures accounted for the direct effect of willpower belief on relationship satisfaction.

Similarity in willpower belief, on the other hand, was unrelated to relationship satisfaction. People’s willpower beliefs did weakly but significantly correlate with the willpower belief of their romantic partner, but the correlation was not moderated by relationship length, suggesting that some assortative mating may be at play (Luo, 2017). However, the degree of similarity in willpower belief did not predict relationship satisfaction, just as similarity in trait self-control or personality traits is typically not associated with relationship quality (Arránz Becker, 2013; Vohs et al., 2011).

### Limitations and future directions

The dyadic data used to test these hypotheses have many strengths, including a large sample size and day-level data on both received and provided support, but still had limitations. One possible complication of the study design was that the various individual difference measures were collected at differing time-points; the willpower belief measure was completed at the end of the two weeks of daily surveys, while the personality trait inventory was completed before the two weeks of surveys. Comparing the predictive strengths of collinear predictors is already statistically difficult (Barni, 2015), and the temporal difference could introduce additional variability that can further interfere with the ability to compare competing predictors. This might explain why, in the current study, only some of the effects of willpower belief remained significant predictors in models with simultaneous predictors – for example, actor effects on relationship satisfaction remained significant, but actor effects on support provision did not, unlike in Francis et al. (2020). However, given that both personality traits and willpower beliefs are moderately stable (Bernecker et al., 2017; Sieber et al., 2019; Wagner et al., 2019), the difference in measurement timepoints across a 14-day period should have only a small impact on the results.

Ultimately, the described associations are correlational. While we considered the daily relationship measures as our outcome variables for the present analyses, we do not know that willpower beliefs are causally affecting relationship satisfaction, support provision, or the receipt of support. It could certainly be the case – instead or also – that the quality of someone’s romantic relationship affects their willpower beliefs. Potentially, someone with lower relationship satisfaction might experiencing more demands or conflicts in their relationship, which may lead them to experience and believe that their willpower is more limited.

Like most relationship research, there are also limitations to the representativeness and generalizability of the sample. Only female–male couples were included in the present study in order to conduct dyadic analyses with distinguishable partners; dynamics of social support may differ among same-gender couples (Umberson et al., 2015). Additionally, associations between willpower belief, support provision, and relationship satisfaction may differ in other cultures, where we see differences in the populations’ willpower beliefs (Savani & Job, 2017; Sun et al., 2019) and ways of exchanging social support (Chen et al., 2015).

Finally, the measures for emotional and instrumental support were single-item measures with binary responses, potentially limiting the scope and granularity of our measurements of support. If social support was instead measured on a continuum to distinguish between days with a single instance of a type of support and multiple instances of that support, we may have been able to better gauge the magnitude of these associations with individual differences. Additionally, the broad framing of the question may have resulted in participants not considering or reporting small every-day behaviours of social support that they regularly provide or receive. More specific and concrete checklists of social support would be interesting to examine in future work (e.g. Egan, 2020). More comprehensive measures may also facilitate differentiation between patterns of emotional and instrumental support.

### Conclusion

The more someone believes that their willpower is limited and depletable, the less likely they are to provide support – both emotional and instrumental – to their romantic partners. The provision of support can itself be effortful, and people with limited willpower beliefs may not always feel that they have the resources to support their partner. Beyond reducing the provision of support, associations between willpower belief and relationship outcomes seem to be largely isolated to the individual. The willpower belief of one’s partner did not have significant impacts on one’s own relationship satisfaction and having a similar willpower belief to one’s romantic partner was also not consequential. On the other hand, those who believed their willpower was limited tended to experience lower relationship satisfaction, and were less likely to receive instrumental support from their partners. Thus, while willpower belief may play a role in the functioning of relationships – particularly relating to the provision of support – our willpower belief likely impacts ourselves more than our romantic partners.

## Supplemental Material

Supplemental Material - My willpower belief and yours: Investigating dyadic associations between willpower beliefs, social support, and relationship satisfaction in couples Supplemental Material for My willpower belief and yours: Investigating dyadic associations between willpower beliefs, social support, and relationship satisfaction in couples by Zoë Francis, Rebekka Weidmann, Janina L. Bühler, Robert P. Burriss, Jenna Wünsche, Alexander Grob and Veronika Job in European Journal of Personality.

## Notes

1. Note that the terms actor effect and partner effect do not mean to imply causality—they merely are used to label whether both measurements were collected from the same individual or across individuals in a couple. The current study only makes correlational observations; willpower beliefs could be the “outcome” that shifts as a result of relationship factors, or vice versa.

2. Data from this longitudinal study have been previously used in a number of published journal articles (Barbaro et al., 2021; Bühler et al., 2020; Gonzalez Avilés et al., 2021; Mund et al., 2020; Nikitin et al., 2021). No other manuscript has analyzed willpower beliefs. One other manuscript using the dataset (Reitz et al., 2022) examined social support as a moderating factor on the association between bereavement and life satisfaction.

3. Reported results of daily relationship satisfaction are from a model including one’s own willpower belief, one’s partner´s willpower belief, and one’s partner’s relationship satisfaction as a covariate. This covariate was included to more closely parallel APIM analyses (including the APIM conducted on global relationship assessment, see Supplemental Materials). Coefficients of willpower belief without the covariate are similar (actor effect *b* = −.08, SE = .02, *t*(822) = −4.01, *p* < .001, partner effect *b* = −.03, SE = .02, *t*(825) = −1.50, *p* = .134).

4. Following our a priori research plan, we conducted an additional analysis to see whether similarity in willpower belief predicted relationship satisfaction specifically on days where couples engaged in joint activities. There was no evidence for this moderation (interaction *b* = −.01, SE = .03, *t*(13786) = −.41, *p* = .683).

## References

[bibr1-08902070231220416] Ackerman R. A. Kenny D. A. (2016). APIMPower: An interactive tool for Actor-Partner Interdependence Model power analysis. Wiley

[bibr2-08902070231220416] Arránz Becker O. (2013). Effects of similarity of life goals, values, and personality on relationship satisfaction and stability: Findings from a two-wave panel study. Personal Relationships, 20(3), 443–461. 10.1111/j.1475-6811.2012.01417.x

[bibr3-08902070231220416] Avivi Y. E. Laurenceau J.-P. Carver C. S. (2009). Linking relationship quality to perceived mutuality of relationship goals and perceived goal progress. Journal of Social and Clinical Psychology, 28(2), 137–164. 10.1521/jscp.2009.28.2.137

[bibr4-08902070231220416] Barbaro N. Weidmann R. Burriss R. P. Wünsche J. Bühler J. L. Shackelford T. K. Grob A. (2021). The (bidirectional) associations between romantic attachment orientations and mate retention behavior in male-female romantic couples. Evolution and Human Behavior, 42(6), 497–506. 10.1016/j.evolhumbehav.2021.04.005

[bibr5-08902070231220416] Barni D. (2015). Relative importance analysis for the study of the family: Accepting the challenge of correlated predictors. TPM - Testing, Psychometrics, Methodology in Applied Psychology, 22(2), 235–250. 10.4473/TPM22.2.5

[bibr6-08902070231220416] Bates D. Mächler M. Bolker B. Walker S. (2015). Fitting linear mixed-effects models using lme4. Journal of Statistical Software, 67, 1–48. 10.18637/jss.v067.i01

[bibr7-08902070231220416] Bernecker K. Herrmann M. Brandstätter V. Job V. (2017). Implicit theories about willpower predict subjective well-being. Journal of Personality, 85(2), 136–150. 10.1111/jopy.12225 26331597

[bibr8-08902070231220416] Bernecker K. Job V. (2015). Beliefs about willpower are related to therapy adherence and psychological adjustment in patients with type 2 diabetes. Basic and Applied Social Psychology, 37(3), 188–195. 10.1080/01973533.2015.1049348

[bibr9-08902070231220416] Bodenmann G. Meuwly N. Germann J. Nussbeck F. W. Heinrichs M. Bradbury T. N. (2015). Effects of stress on the social support provided by men and women in intimate relationships. Psychological Science, 26(10), 1584–1594. 10.1177/0956797615594616 26341561

[bibr10-08902070231220416] Brunstein J. C. Dangelmayer G. Schultheiss O. C. (1996). Personal goals and social support in close relationships: Effects on relationship mood and marital satisfaction. Journal of Personality and Social Psychology, 71(5), 1006–1019. 10.1037//0022-3514.71.5.1006

[bibr11-08902070231220416] Bühler J. L. Weidmann R. Wünsche J. Burriss R. P. Grob A. (2020). Daily responsiveness, expectations, and self-disclosure: How the average levels and within-person variability of three relationship components mediate personality–relationship transactions in romantic couples. European Journal of Personality, 34(3), 367–392. 10.1002/per.2255

[bibr12-08902070231220416] Cameron C. D. Hutcherson C. A. Ferguson A. M. Scheffer J. A. Hadjiandreou E. Inzlicht M. (2019). Empathy is hard work: People choose to avoid empathy because of its cognitive costs. Journal of Experimental Psychology: General, 148(6), 962–976. 10.1037/xge0000595 30998038

[bibr13-08902070231220416] Campbell L. Stanton S. C. E. (2015). Actor-partner interdependence model. The Encyclopedia of Clinical Psychology, 1, 1–7. 10.1002/9781118625392.wbecp467

[bibr14-08902070231220416] Candel O. S. Turliuc M. N. (2019). Insecure attachment and relationship satisfaction: A meta-analysis of actor and partner associations. Personality and Individual Differences, 147, 190–199. 10.1016/j.paid.2019.04.037

[bibr15-08902070231220416] Chen J. M. Kim H. S. Sherman D. K. Hashimoto T. (2015). Cultural differences in support provision: The importance of relationship quality. Personality and Social Psychology Bulletin, 41(11), 1575–1589. 10.1177/0146167215602224 26351274

[bibr16-08902070231220416] Collins N. L. Kane H. S. Metz M. A. Cleveland C. Khan C. Winczewski L. Bowen J. Prok T. (2014). Psychological, physiological, and behavioral responses to a partner in need: The role of compassionate love. Journal of Social and Personal Relationships, 31(5), 601–629. 10.1177/0265407514529069

[bibr17-08902070231220416] Cutrona C. E. Russell D. W. (2017). Autonomy promotion, responsiveness, and emotion regulation promote effective social support in times of stress. Current Opinion in Psychology, 13, 126–130. 10.1016/j.copsyc.2016.07.002 28813282

[bibr18-08902070231220416] de Ridder D. T. D. Lensvelt-Mulders G. Finkenauer C. Stok F. M. Baumeister R. F. (2012). Taking stock of self-control: A meta-analysis of how trait self-control relates to a wide range of behaviors. Personality and Social Psychology Review: An Official Journal of the Society for Personality and Social Psychology, Inc, 16(1), 76–99. 10.1177/1088868311418749 21878607

[bibr19-08902070231220416] Devoldre I. Davis M. H. Verhofstadt L. L. Buysse A. (2010). Empathy and social support provision in couples: Social support and the need to study the underlying processes. Journal of Psychology, 144(3), 259–284. 10.1080/00223981003648294 20461931

[bibr20-08902070231220416] Di Maio S. Keller J. Job V. Felsenberg D. Ertel W. Schwarzer R. Knoll N. (2020). Health demands moderate the link between willpower beliefs and physical activity in patients with knee osteoarthritis. International Journal of Behavioral Medicine, 27(4), 406–414. 10.1007/s12529-020-09865-w 32162213 PMC7359122

[bibr21-08902070231220416] Egan R. P. (2020). Development and validation of the instrumental support inventory for spouses. Notre Dame.

[bibr22-08902070231220416] Faul F. Erdfelder E. Buchner A. Lang A.-G. (2009). Statistical power analyses using G*Power 3.1: Tests for correlation and regression analyses. Behavior Research Methods, 41(4), 1149–1160. 10.3758/BRM.41.4.1149 19897823

[bibr23-08902070231220416] Feeney B. C. Collins N. L. (2015). A new look at social support: A theoretical perspective on thriving through relationships. Personality and Social Psychology Review, 19(2), 113–147. 10.1177/1088868314544222 25125368 PMC5480897

[bibr24-08902070231220416] Ferguson A. M. Cameron C. D. Inzlicht M. (2021). When does empathy feel good? Current Opinion in Behavioral Sciences, 39, 125–129. 10.1016/j.cobeha.2021.03.011

[bibr25-08902070231220416] Finkel E. J. Fitzsimons G. M. VanDellen M. R. (2016). Self-regulation as a transactive process: Reconceptualizing the unit of analysis for goal setting, pursuit, and outcomes. In Vohs K. D. Baumeister R. F. (eds), Handbook of self-regulation (3rd ed., pp. 1–36). Guilford Press.

[bibr26-08902070231220416] Fitzsimons G. M. Finkel E. J. VanDellen M. R. (2015). Transactive goal dynamics. Psychological Review, 122(4), 648–673. 10.1037/a0039654 26437147

[bibr27-08902070231220416] Fraley R. C. Heffernan M. E. Vicary A. M. Brumbaugh C. C. (2011). The experiences in close relationships -relationship Structures questionnaire: A method for assessing attachment orientations across relationships. Psychological Assessment, 23(3), 615–625. 10.1037/a0022898 21443364

[bibr28-08902070231220416] Francis Z. Job V. (2018). Lay theories of willpower. Social and Personality Psychology Compass, 12(4), Article e12381. 10.1111/spc3.12381

[bibr29-08902070231220416] Francis Z. Job V. (2021). Intended responses to romantic partners’ annoying behaviours vary with willpower beliefs. British Journal of Psychology, 112(2), 549–564. 10.1111/bjop.12475 32997343

[bibr30-08902070231220416] Francis Z. Mata J. Flückiger L. Job V. (2021). Morning resolutions, evening disillusions: Theories of willpower affect how health behaviours change across the day. European Journal of Personality, 35(3), 398–415. 10.1177/0890207020962304

[bibr31-08902070231220416] Francis Z. Sieber V. Job V. (2020). You seem tired, but so am I: Willpower theories and intention to provide support in romantic relationships. Journal of Social and Personal Relationships, 37(3), 738–757. 10.1177/0265407519877238

[bibr32-08902070231220416] Girme Y. U. Overall N. C. Faingataa S. (2014). “Date nights” take two: The maintenance function of shared relationship activities. Personal Relationships, 21(1), 125–149. 10.1111/pere.12020

[bibr33-08902070231220416] Gollwitzer P. M. Sheeran P. (2006). Implementation intentions and goal achievement: A meta-analysis of effects and processes. Advances in Experimental Social Psychology, 38(06), 69–119. 10.1016/S0065-2601(06)38002-1

[bibr34-08902070231220416] Gonzalez Avilés T. Burriss R. P. Weidmann R. Bühler J. L. Wünsche J. Grob A. (2021). Committing to a romantic partner: Does attractiveness matter? A dyadic approach. Personality and Individual Differences, 176, 110765. 10.1016/j.paid.2021.110765

[bibr35-08902070231220416] Gosnell C. L. Gable S. L. (2017). You deplete me: Impacts of providing positive and negative event support on self-control. Personal Relationships, 24(3), 598–622. 10.1111/pere.12200

[bibr36-08902070231220416] Gray J. S. Dunlop W. L. (2019). Structure and measurement invariance of adult romantic attachment. Journal of Personality Assessment, 101(2), 171–180. 10.1080/00223891.2017.1391274 29206485

[bibr37-08902070231220416] Gustavson K. Røysamb E. Borren I. Torvik F. A. Karevold E. (2016). Life satisfaction in close relationships: Findings from a longitudinal study. Journal of Happiness Studies, 17(3), 1293–1311. 10.1007/s10902-015-9643-7

[bibr38-08902070231220416] Iida M. Parris Stephens M. A. Rook K. S. Franks M. M. Salem J. K. (2010). When the going gets tough, does support get going? Determinants of spousal support provision to type 2 diabetic patients. Personality and Social Psychology Bulletin, 36(6), 780–791. 10.1177/0146167210369897 20445023

[bibr39-08902070231220416] Iida M. Seidman G. Shrout P. E. Fujita K. Bolger N. (2008). Modeling support provision in intimate relationships. Journal of Personality and Social Psychology, 94(3), 460–478. 10.1037/0022-3514.94.3.460 18284292

[bibr40-08902070231220416] Jędrzejczyk J. Zajenkowski M. (2020). Who believes in nonlimited willpower? In search of correlates of implicit theories of self-control. Psychological Reports, 123(2), 281–299. 10.1177/0033294118809936 30398408

[bibr41-08902070231220416] Job V. Bernecker K. Miketta S. Friese M. (2015). Implicit theories about willpower predict the activation of a rest goal following self-control exertion. Journal of Personality and Social Psychology, 109(4), 694–706. 10.1037/pspp0000042 26075793

[bibr42-08902070231220416] Job V. Dweck C. S. Walton G. M. (2010). Ego depletion—is it all in your head? Implicit theories about willpower affect self-regulation. Psychological Science, 21(11), 1686–1693. 10.1177/0956797610384745 20876879

[bibr43-08902070231220416] Job V. Sieber V. Rothermund K. Nikitin J. (2018). Age differences in implicit theories about willpower: Why older people endorse a nonlimited theory. Psychology and Aging, 33(6), 940–952. 10.1037/pag0000285 30198732

[bibr44-08902070231220416] Jocz P. Stolarski M. Jankowski K. S. (2018). Similarity in chronotype and preferred time for sex and its role in relationship quality and sexual satisfaction. Frontiers in Psychology, 9, 443–512. 10.3389/fpsyg.2018.00443 29670559 PMC5893780

[bibr45-08902070231220416] John O. P. Srivastava S. (1999). The Big-Five trait taxonomy: History, measurement, and theoretical perspectives. In Pervin L. A. John O. P. (eds), Handbook of personality: Theory and research (p. 102–138). Guilford Press.

[bibr46-08902070231220416] Kenny D. A. Kashy D. A. Cook W. L. (2006). Dyadic data analysis. Guilford Press.

[bibr47-08902070231220416] Klinger J. Scholer A. Hui C. M. Molden D. C. (2018). Effortful experiences of self-control foster lay theories that self-control is limited. Journal of Experimental Social Psychology, 78, 1–13. 10.1016/j.jesp.2018.04.006

[bibr48-08902070231220416] Konze A.-K. Rivkin W. Schmidt K.-H. (2018). Implicit theories about willpower as a moderator of the adverse effect of daily self-control demands on need for recovery. Zeitschrift für Arbeitswissenschaft, 72(1), 61–70. 10.1007/s41449-017-0062-y

[bibr49-08902070231220416] Kuznetsova A. Brockhoff P. Christensen R. H. B. (2017). lmerTest Package: Tests in linear mixed effects models. Journal of Statistical Software, 82(13), 1–26. 10.18637/jss.v082.i13

[bibr50-08902070231220416] Leikas S. Ilmarinen V. J. Verkasalo M. Vartiainen H. L. Lönnqvist J. E. (2018). Relationship satisfaction and similarity of personality traits, personal values, and attitudes. Personality and Individual Differences, 123, 191–198. 10.1016/j.paid.2017.11.024

[bibr51-08902070231220416] Levesque C. Lafontaine M. F. Caron A. Flesch J. L. Bjornson S. (2014). Dyadic empathy, dyadic coping, and relationship satisfaction: A dyadic model. Europe's Journal of Psychology, 10(1), 118–134. 10.5964/ejop.v10i1.697

[bibr82-08902070231220416] Li T. Chan D. K. S. (2012). How anxious and avoidant attachment affect romantic relationship quality differently: A meta‐analytic review. European Journal of Social Psychology, 42(4), 406–419. 10.1002/ejsp.1842

[bibr52-08902070231220416] Luo S. (2017). Assortative mating and couple similarity: Patterns, mechanisms, and consequences. Social and Personality Psychology Compass, 11(8), Article e12337. 10.1111/spc3.12337

[bibr53-08902070231220416] Malouff J. M. Thorsteinsson E. B. Schutte N. S. Bhullar N. Rooke S. E. (2010). The five-factor model of personality and relationship satisfaction of intimate partners: A meta-analysis. Journal of Research in Personality, 44(1), 124–127. 10.1016/j.jrp.2009.09.004

[bibr54-08902070231220416] McNeish D. (2018). Thanks coefficient alpha, we’ll take it from here. Psychological Methods, 23(3), 412–433. 10.1037/met0000144 28557467

[bibr55-08902070231220416] Molero F. Shaver P. R. Fernández I. Recio P. (2017). Attachment insecurities, life satisfaction, and relationship satisfaction from a dyadic perspective: The role of positive and negative affect. European Journal of Social Psychology, 47(3), 337–347. 10.1002/ejsp.2276

[bibr56-08902070231220416] Moreira J. M. de Fátima Silva M. Moleiro C. Aguiar P. Andrez M. Bernardes S. Afonso H. (2003). Perceived social support as an offshoot of attachment style. Personality and Individual Differences, 34(3), 485–501. 10.1016/S0191-8869(02)00085-5

[bibr57-08902070231220416] Morgan P. Love H. A. Durtschi J. May S. (2018). Dyadic causal sequencing of depressive symptoms and relationship satisfaction in romantic partners across four years. American Journal of Family Therapy, 46(5), 486–504. 10.1080/01926187.2018.1563004

[bibr58-08902070231220416] Mukhopadhyay A. Johar G. V. (2005). Where there is a will, is there a way? Effects of lay theories of self-control on setting and keeping resolutions. Journal of Consumer Research, 31(4), 779–786. 10.1086/426611

[bibr59-08902070231220416] Mund M. Weidmann R. Wrzus C. Johnson M. D. Bühler J. L. Burriss R. P. Wünsche J. Grob A. (2020). Loneliness is associated with the subjective evaluation of but not daily dynamics in partner relationships. International Journal of Behavioral Development, 46(1), 28–38. 10.1177/0165025420951246

[bibr83-08902070231220416] Napolitano C. M. Job V. (2018). Assessing the implicit theory of willpower for strenuous mental activities scale: Multigroup, across-gender, and cross-cultural measurement invariance and convergent and divergent validity. Psychological Assessment, 30(8), 1049. 10.1037/pas0000557 29781663

[bibr60-08902070231220416] Neff L. A. Karney B. R. (2005). Gender differences in social support : A question of skill or responsiveness? Journal of Personality and Social Psychology, 88(1), 79–90. 10.1037/0022-3514.88.1.79 15631576

[bibr61-08902070231220416] Nikitin J. Wünsche J. Bühler J. L. Weidmann R. Burriss R. P. Grob A. (2021). Interdependence of approach and avoidance goals in romantic couples over days and months. Journals of Gerontology Series B: Psychological Sciences and Social Sciences, 76(7), 1251–1263. 10.1093/geronb/gbaa149 32882014

[bibr62-08902070231220416] Rammstedt B. Danner D. (2016). Die Facettenstruktur des Big Five Inventory (BFI): Validierung für die deutsche Adaptation des BFI. Diagnostica, 63(1), 70–84. 10.1026/0012-1924/a000161

[bibr63-08902070231220416] R Core Team (2021). R: A language and environment for statistical computing. R Foundation for Statistical Computing.

[bibr64-08902070231220416] Reitz A. K. Weidmann R. Wünsche J. Bühler J. L. Burriss R. P. Grob A. (2022). In good times and in bad: A longitudinal analysis of the impact of bereavement on self-esteem and life satisfaction in couples. European Journal of Personality, 36(4), 616–639. 10.1177/08902070211054896

[bibr65-08902070231220416] Righetti F. Finkenauer C. (2011). If you are able to control yourself, I will trust you: The role of perceived self-control in interpersonal trust. Journal of Personality and Social Psychology, 100(5), 874–886. 10.1037/a0021827 21319912

[bibr66-08902070231220416] Righetti F. Impett E. (2017). Sacrifice in close relationships: Motives, emotions, and relationship outcomes. Social and Personality Psychology Compass, 11(10), 1–11. 10.1111/spc3.12342

[bibr67-08902070231220416] Sakaluk J. K. Fisher A. N. Kilshaw R. E. (2021). Dyadic measurement invariance and its importance for replicability in romantic relationship science. Personal Relationships, 28(1), 190–226. 10.1111/pere.12341

[bibr68-08902070231220416] Savani K. Job V. (2017). Reverse ego-depletion: Acts of self-control can improve subsequent performance in Indian cultural contexts. Journal of Personality and Social Psychology, 113(4), 589–607. 10.1037/pspi0000099 28581300

[bibr69-08902070231220416] Scheffer J. A. Cameron C. D. Inzlicht M. (2021). Caring is costly: People avoid the cognitive work of compassion. Journal of Experimental Psychology: General, 151(1), 172–196. 10.1037/xge0001073 34410802

[bibr70-08902070231220416] Sened H. Lavidor M. Lazarus G. Bar-Kalifa E. Rafaeli E. Ickes W. (2017). Empathic accuracy and relationship satisfaction: A meta-analytic review. Journal of Family Psychology: JFP: journal of the Division of Family Psychology of the American Psychological Association (Division 43), 31(6), 742–752. 10.1037/fam0000320 28394141

[bibr71-08902070231220416] Shrout P. E. Herman C. M. Bolger N. (2006). The costs and benefits of practical and emotional support on adjustment: A daily diary study of couples experiencing acute stress. Personal Relationships, 13(1), 115–134. 10.1111/j.1475-6811.2006.00108.x

[bibr72-08902070231220416] Sieber V. Flückiger L. Mata J. Bernecker K. Job V. (2019). Autonomous goal striving promotes a nonlimited theory about willpower. Personality and Social Psychology Bulletin, 45(8), 1295–1307. 10.1177/0146167218820921 30654723

[bibr73-08902070231220416] Sniehotta F. F. (2009). Towards a theory of intentional behaviour change: Plans, planning, and self-regulation. British Journal of Health Psychology, 14(Pt 2), 261–273. 10.1348/135910708X389042 19102817

[bibr74-08902070231220416] Sullivan K. T. Pasch L. A. Johnson M. D. Bradbury T. N. (2010). Social support , problem solving , and the longitudinal course of newlywed marriage. Journal of Personality and Social Psychology, 98(4), 631–644. 10.1037/a0017578 20307134

[bibr75-08902070231220416] Sun X. Cortina K. S. Miller K. F. Ning H. (2019). Willpower as cultural construct: Do Chinese students believe less in its depletion? Frontiers in Psychology, 10, 988–997. 10.3389/fpsyg.2019.00988 31133929 PMC6512892

[bibr76-08902070231220416] Taylor S. E. (2011). Social support: A review. In Friedman H. S. (Ed.), Oxford handbook of health psychology (pp. 192–217). Oxford University Press

[bibr77-08902070231220416] Umberson D. Thomeer M. B. Lodge A. C. (2015). Intimacy and emotion work in lesbian, gay, and heterosexual relationships. Journal of Marriage and Family, 77(2), 542–556. 10.1111/jomf.12178 25814771 PMC4370347

[bibr78-08902070231220416] Verhofstadt L. L. Buysse A. Ickes W. (2007). Social support in couples: An examination of gender differences using self-report and observational methods. Sex Roles, 57(3-4), 267–282. 10.1007/s11199-007-9257-6

[bibr79-08902070231220416] Vohs K. D. Finkenauer C. Baumeister R. F. (2011). The sum of friends’ and lovers’ self-control scores predicts relationship quality. Social Psychological and Personality Science, 2(2), 138–145. 10.1177/1948550610385710

[bibr80-08902070231220416] Wagner J. Lüdtke O. Robitzsch A. (2019). Does personality become more stable with age? Disentangling state and trait effects for the big five across the life span using local structural equation modeling. Journal of Personality and Social Psychology, 116(4), 666–680. 10.1037/pspp0000203 30714756

[bibr81-08902070231220416] Weidmann R. Schönbrodt F. D. Ledermann T. Grob A. (2017). Concurrent and longitudinal dyadic polynomial regression analyses of Big Five traits and relationship satisfaction: Does similarity matter? Journal of Research in Personality, 70, 6–15. 10.1016/j.jrp.2017.04.003

[bibr84-08902070231220416] Wu S. Crespi C. M. Wong W. K. (2012). Comparison of methods for estimating the intraclass correlation coefficient for binary responses in cancer prevention cluster randomized trials. Contemporary Clinical Trials, 33(5), 869–880. 10.1016/j.cct.2012.05.004 22627076 PMC3426610

