# Supplementary material for: My willpower belief and yours: Investigating dyadic associations between willpower beliefs, social support, and relationship satisfaction in couples
Source: Eur J Pers. 2023 Dec 14;38(5):778–92. doi: 10.1177/08902070231220416 (PMC13021047; doi:10.1177/08902070231220416)
Supplement: Supplemental Material - My willpower belief and yours: Investigating dyadic associations between willpower beliefs, social support, and relationship satisfaction in couples [file sj-pdf-1-erp-10.1177_08902070231220416.pdf]

Supplemental Materials for

My Willpower Belief and Yours:

Investigating Actor, Partner, and Similarity Effects of Willpower Beliefs on Social Support and Relationship Satisfaction in Couples

Table of Contents

Global Measure of Relationship Satisfaction	2
Measure and Analysis Plan	2
Associations with Willpower Belief using the Actor-Partner Interdependence Model	2
Figure S1. *APIM of Associations between Willpower belief and global relationship satisfaction*	3
Similarity Effects (Dyadic Response Surface Analysis)	3
Table S1. *Coefficients from the dyadic response surface analysis*	4
Figure S2. *The dyadic response surface analysis path model*	5
Exploratory Mediation Model via Affect	6
Associations between Relationship Satisfaction and Support Receipt	7
Structural Equation Model Fit for Willpower Belief Measure	8
Associations with Two Subscales of Willpower Belief Measure	

Global Measure of Relationship Satisfaction

Measure and Analysis Plan

In addition to being measured daily, wholistic relationship satisfaction was measured on the last day of the two-week wave, using the seven-item Relationship Assessment scale (Hendrick, 1988; Sander & Böcker, 1993). Items were measured on a five-point Likert scale and averaged. The highest-loading items were, “In general, how satisfied are you with your relationship?” and “How good is your relationship compared to most?”. The scale had good internal reliability, α = .88.

Analyses of the global Relationship Assessment scale were conducted by creating structural equation models using the *lavaan* package version 0.6-9 (Rosseel, 2012). Following the APIM, we modelled pathways for both actor and partner effects, and a pathway for covariance between the dependent variable of the two partners.

Associations with Willpower Belief using the Actor-Partner Interdependence Model

To investigate both the actor effects and partner effects of willpower belief on global relationship satisfaction[1], we conducted a structural equation model following the actor-partner interdependence model (APIM; Figure S1). We compared models with equality constraints for the men’s and women’s pathways to models without equality constraints, comparing the models using χ^2 tests and comparing the models’ BICs (Horne & Johnson, 2019; Kenny et al., 2006). Finding no differences in the model fit, we report the pathways from the more parsimonious constrained model, with single parameter estimates for the actor effect (regardless of gender) and

[1] Our *a priori* research plan hypothesized that willpower belief may also affect global relationship commitment, in addition to global relationship satisfaction. Willpower belief did not predict commitment as measured with the 9-item investment model scale (Grau et al., 2001; Rusbult et al., 1998), neither as an actor effect (B = −0.004, z = 0.112, p = .91) nor as a partner effect (B = −0.004, z = 0.100, p = .92)

for the partner effect (regardless of gender). This final model had good fit ($\chi^2(2) = 2.69$, RMSEA = 0.036, 90% CI [0.00, 0.094], TLI = 0.983).

Figure S1

APIM of Associations between Willpower Belief and Global Relationship Satisfaction

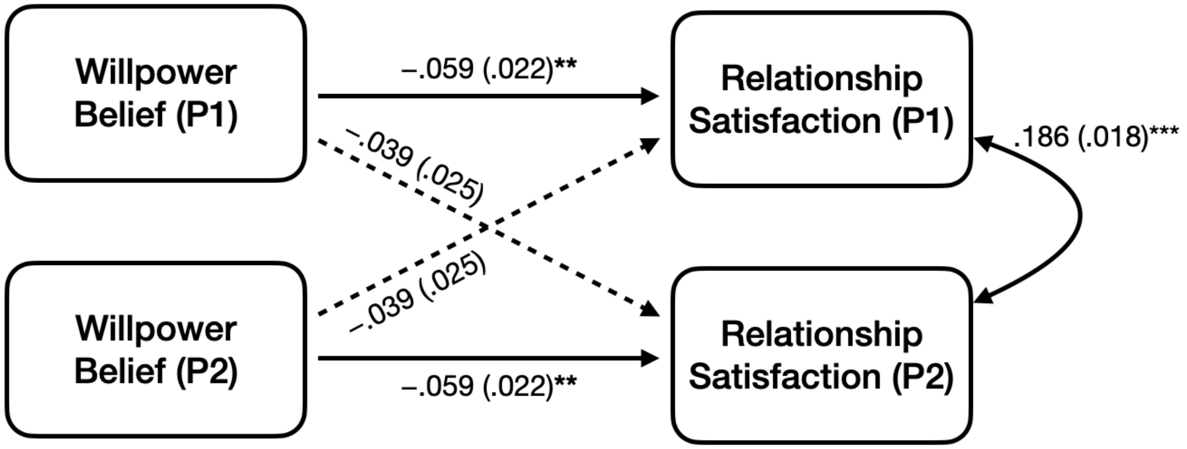

Note. Model was constrained so that both actor effects and both partner effects were equivalent. Dashed lines are not statistically significant at $\alpha < .01$. ***$p < .001$; **$p < .01$.

Consistent with the findings of daily relationship satisfaction, overall relationship assessment was predicted by one's own willpower belief (actor effect, $b = -0.059$, $SE = 0.022$, $z = -2.67$, $p = .008$), with those with more limited willpower beliefs reporting poorer relationship quality. The willpower belief of one's partner did not significantly affect relationship quality (partner effect, $b = -0.039$, $SE = 0.025$, $z = -1.57$, $p = .12$). The relationship quality of the two partners were also associated ($b = 0.186$, $SE = 0.018$, $z = 10.38$, $p < .001$).

Similarity Effects (Dyadic Response Surface Analysis)

We conducted a dyadic response surface analysis following the analysis procedure described in Schönbrodt et al., 2018. We conducted structural equation models, using the R

lavaan package's *sem* function, with the following predictors: willpower belief of each partner, willpower belief^2 of each partner, and the interaction term of the two willpower beliefs (see Figure S2). This analysis, like the analysis described in the main manuscript, found no evidence for the similarity hypothesis (both interaction terms $p > .19$; see Table S1). More rudimentarily, conducting a multi-level model predicting global relationship satisfaction scores nested within couple, the absolute value of the difference between willpower theories did not predict global relationship satisfaction ($b = -0.03$, $SE = 0.04$, $t(545) = -0.83$, $p = .405$).

Table S1

Coefficients from the Dyadic Response Surface Analysis

	**Coefficient**	**b (SE)**	**t-value and p-value**
b1f	Female actor effect (X)	-0.017 (0.032)	-0.520, p = .603
b2f	Male partner effect (Y)	-0.076 (0.038)	-2.031, p = .042
b3f	Female actor effect (X^2)	0.023 (0.027)	0.847, p = .397
b4f	Interaction: Female X * Male Y	0.059 (0.046)	1.295, p = .195
b5f	Male partner effect (Y^2)	-0.056 (0.033)	-1.697, p = .090
b1m	Female partner effect (X)	-0.023 (0.034)	-0.689, p = .491
b2m	Male actor effect (Y)	-0.098 (0.033)	-2.991, p = .003
b3m	Female partner effect (X^2)	-0.065 (0.031)	-2.097, p = .036
b4m	Interaction: Female X * Male Y	0.009 (0.041)	0.223, p = .824
b5m	Male actor effect (Y^2)	0.032 (0.029)	1.085, p = .278

Note. Coefficients b4f and b4m represent similarity effects. For visualization of these pathways, see Figure S2 (below).

Figure S2

The Dyadic Response Surface Analysis Path Model

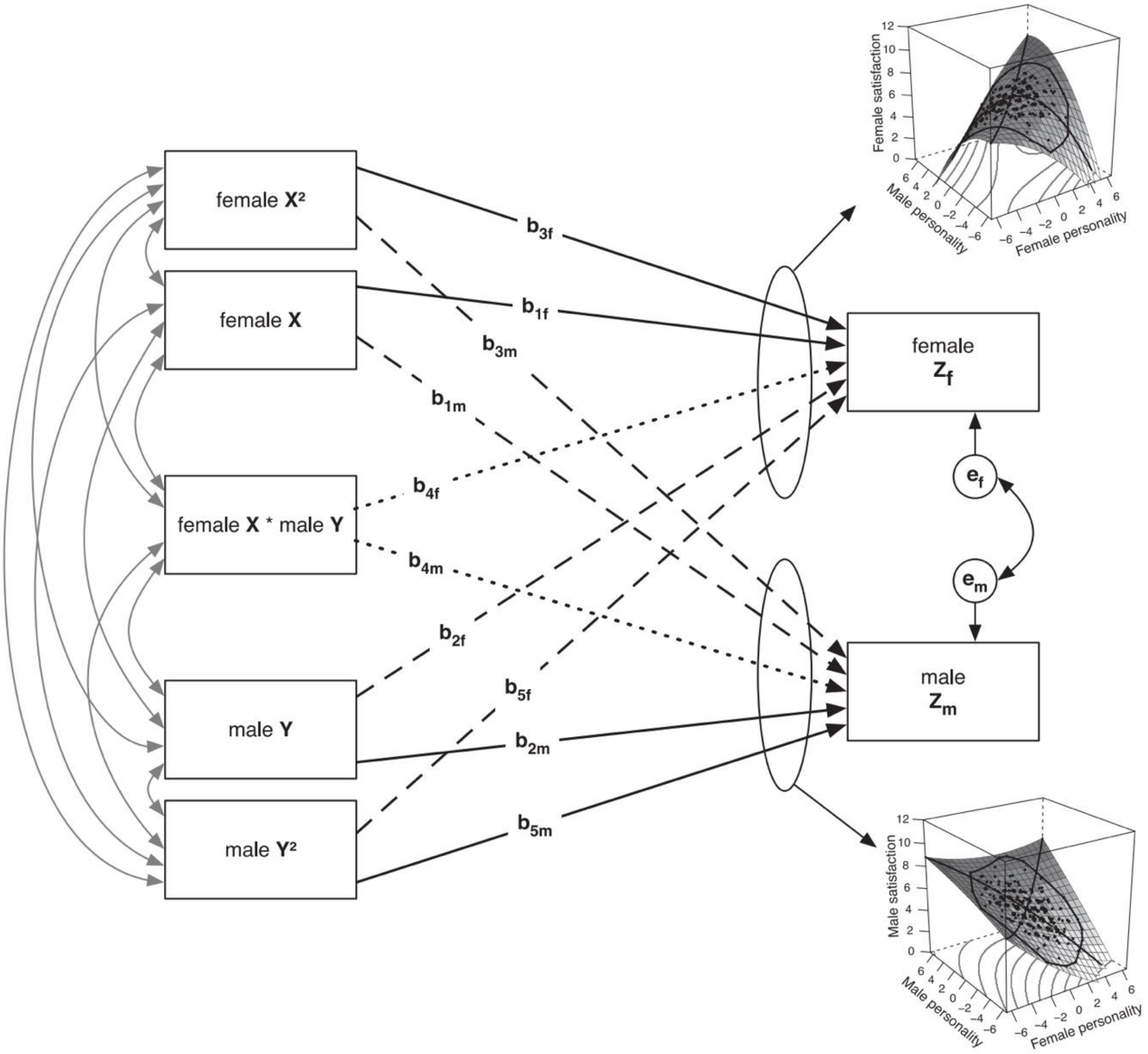

Note. Black solid paths are actor effects, dashed paths are partner effects, and the dotted paths are statistical partner interactions. Intercepts are not displayed. Figure available at https://osf.io/ftsrd/, under a CC-BY4.0 licence.

Figure is reproduced, without modifications, from:

Schönbrodt, F. D., Humberg, S., & Nestler, S. (2018). Testing similarity effects with dyadic response surface analysis. *European Journal of Personality*, *32*(6), 627–641. https://doi.org/10.1002/per.2169.

Exploratory Mediation Via Affect

The direct effect of willpower belief on one's own daily relationship satisfaction was mediated by participants mean level of affect (Figure S3). Affect was measured via the German PANAS, by averaging positive states and reverse-scored negative states (Krohne et al., 1996; Watson et al., 1988). The confidence interval for the indirect effect was calculated via Monte Carlo bootstrapping (Segil & Preacher, 2008).

Figure S3

Exploratory Mediation Model

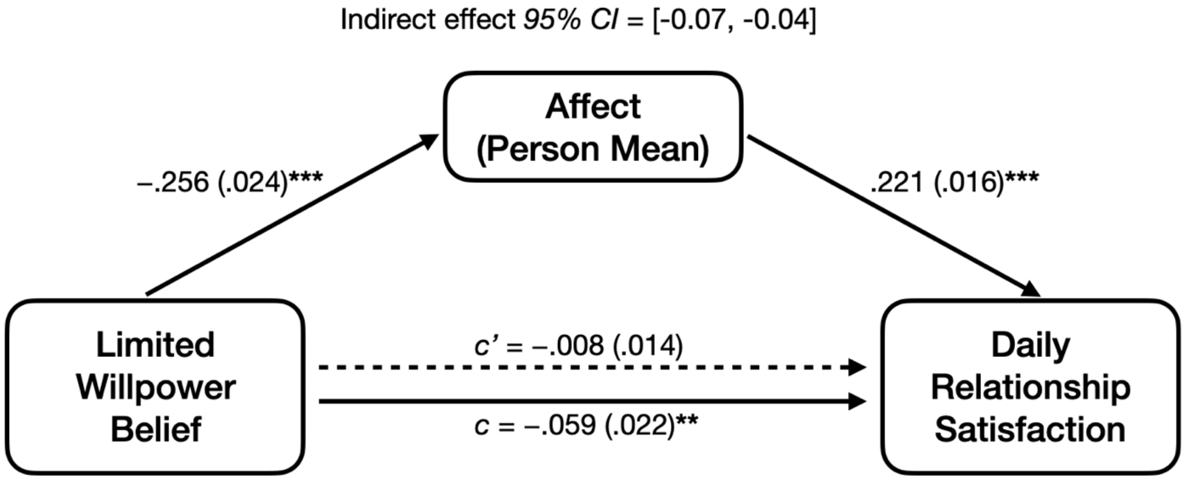

Associations between Relationship Satisfaction and Support Receipt

Correlational relationships between relationship satisfaction and social support are complex. For example, someone who is unhappy and struggling is likely to report lower relationship satisfaction and also will likely receive more social support (resulting in a negative relationship between relationship satisfaction and social support), while simultaneously someone who receives social support from their partner will feel more supported, closer, and report higher relationship satisfaction (a positive relationship). Within-subject, participants reported higher relationship satisfaction on days where they received social support—either instrumental or social support ($b = 0.19$, $SE = 0.02$, $t(14923) = 10.39$, $p < .001$, $r = 0.08$; emotional support $b = 0.20$, $SE = 0.02$, $t(14834) = 9.81$, $p < .001$, $r = 0.08$). But, between-subject, participants who were more satisfied with their relationships (on average) received support on a lower proportion of days (instrumental $r(1074) = -0.08$, $p = .01$; and $r(1080) = -0.09$, $p = .003$ for emotional support). This is consistent with past research that describes how the receipt of support is associated with both positive and negative outcomes, including decreased self-esteem and self-efficacy (Bolger et al., 2000; Gleason et al., 2008; Mcclure et al., 2014). Particularly when examined as correlations, receiving social support more frequently is not necessarily a sign of higher relationship quality

Loadings and Measurement Invariance for Willpower Measure

While initial estimates of reliability (i.e., Cronbach's alpha) suggested adequate internal reliability, the fit statistics for the configural dyadic 12-item willpower measure scale were not adequate (χ^2(239, baseline 276) = 3128, p < .001, CFI = .5014, TLI = .427, RMSEA = .149) and some items had quite low loadings on the single factor (e.g., < .4 for men and women). A two-factor model was a better fit, with the items falling along the lines of the two known subscales: *Strenuous Mental Activity* and *Resisting Temptations* (final configural structural equation model in Figure S4, χ^2 (145, 190) = 180.38, p < .001, CFI = .94, TLI = .91, RMSEA = .053).

The Strenuous Mental Activity subscale (6 items) has been previously validated and found to have no measurement invariance (Napolitano & Job, 2018). Following Napolitano and Job (2018), we included a methods factor in the structural model to account for the three reverse-scored items. The resulting dyadic configural model for the Strenuous Mental Activity subscale had good fit (χ^2(42, 66) = 134.90, p < .001, CFI = .974, TLI = .960, RMSEA = .06). We thus used the average of these six items for our analysis of the Strenuous Mental Activity subscale, below.

The configural models of the Resisting Temptations subscale with all six items was initially not acceptable. While adding a methods factor for the three reverse-scored item improved model fit, it was still not adequate (with methods factor, TLI = .905, CFI = .940, RMSEA = .075). Two items had low loadings (item 9, loadings .34 for women and .24 for men; item 11, loadings .49 and .33). After removing these two items, the dyadic configural model for the Resisting Temptations subscale had excellent fit (χ^2(15,28)= 19.364, p = .20, TLI = .995, CFI = .997, RMSEA = .022, no methods factor). We thus used the average of the remaining four items for our analysis of the Resisting Temptations subscale.

Figure S4

Structural Equation Model

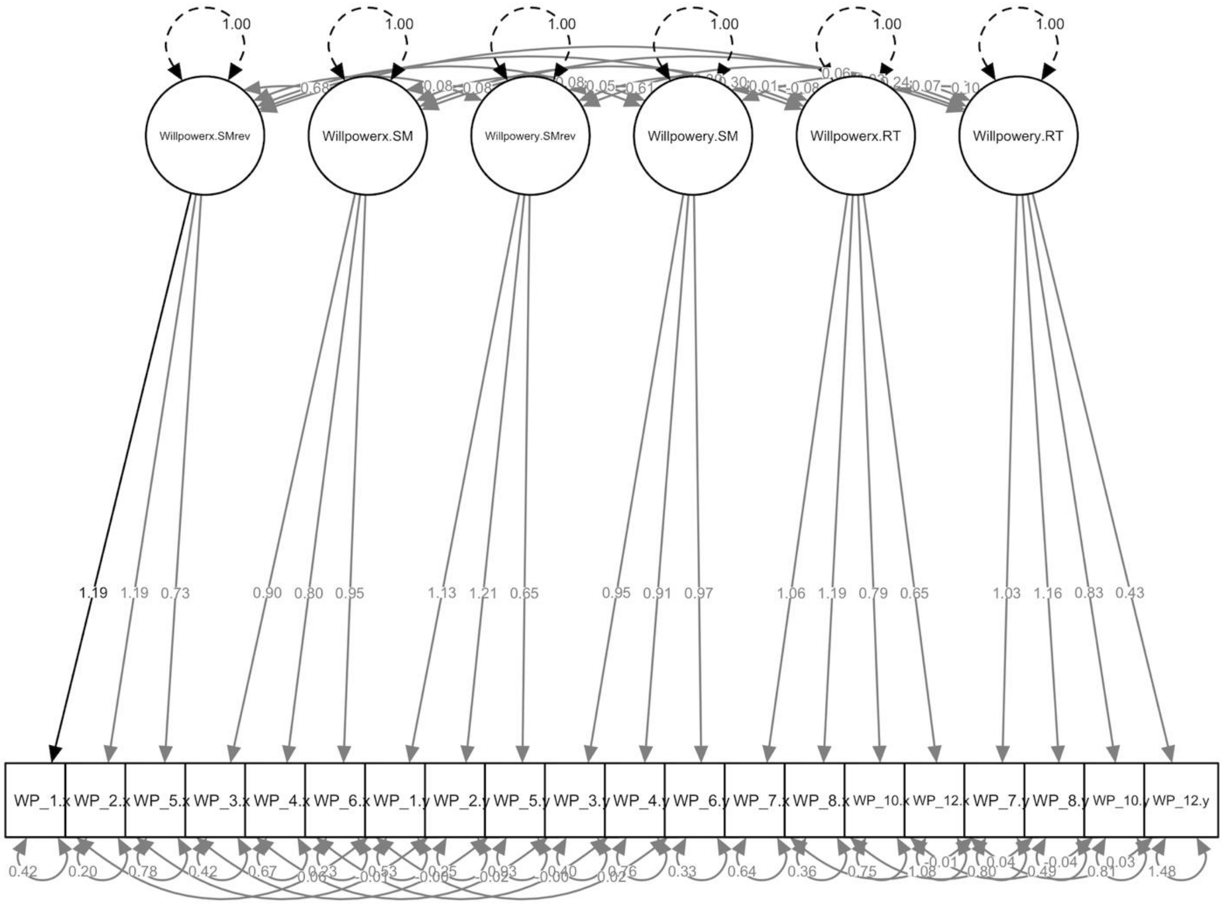

Note. See Table S2 below for loadings

Table S2

Factor Loadings for the Structural Equation Model of the Willpower Beliefs Measure

Latent Factor	Indicator	Loading	SE	Z	p-value	Std. Loading	Intercept
Will.x.SM	*WP_1.x*	*1.106*	*0.049*	*22.75*	*< .001*	*0.819*	*4.17*
Will.x.SM	*WP_2.x*	*1.086*	*0.045*	*24.08*	*< .001*	*0.851*	*4.08*
Will.x.SM	*WP_3.x*	*0.783*	*0.043*	*18.34*	*< .001*	*0.703*	*3.44*
Will.x.SM	*WP_4.x*	*0.712*	*0.046*	*15.64*	*< .001*	*0.623*	*3.40*
Will.x.SM	*WP_5.x*	*0.783*	*0.044*	*17.69*	*< .001*	*0.685*	*3.88*
Will.x.SM	*WP_6.x*	*0.794*	*0.040*	*19.42*	*< .001*	*0.734*	*3.47*
Willpowerx.RT	*WP_7.x*	*1.056*	*0.050*	*21.10*	*< .001*	*0.798*	*3.20*
Willpowerx.RT	*WP_8.x*	*1.189*	*0.048*	*24.55*	*< .001*	*0.893*	*3.33*
Willpowerx.RT	*WP_10.x*	*0.789*	*0.046*	*16.99*	*< .001*	*0.673*	*3.05*
Willpowerx.RT	*WP_12.x*	*0.654*	*0.051*	*12.76*	*< .001*	*0.532*	*3.49*
Willpowery.SM	*WP_1.y*	*0.871*	*0.054*	*16.25*	*< .001*	*0.650*	*3.96*
Willpowery.SM	*WP_2.y*	*0.947*	*0.051*	*18.67*	*< .001*	*0.724*	*3.91*
Willpowery.SM	*WP_3.y*	*0.905*	*0.043*	*21.16*	*< .001*	*0.792*	*3.41*
Willpowery.SM	*WP_4.y*	*0.840*	*0.050*	*16.79*	*< .001*	*0.668*	*3.49*
Willpowery.SM	*WP_5.y*	*0.582*	*0.049*	*11.85*	*< .001*	*0.501*	*3.82*
Willpowery.SM	*WP_6.y*	*0.883*	*0.042*	*20.92*	*< .001*	*0.786*	*3.45*
Willpowery.RT	*WP_7.y*	*1.027*	*0.055*	*18.67*	*0.001*	*0.754*	*3.01*
Willpowery.RT	*WP_8.y*	*1.153*	*0.053*	*21.59*	*< .001*	*0.855*	*3.11*
Willpowery.RT	*WP_10.y*	*0.832*	*0.050*	*16.59*	*< .001*	*0.832*	*2.88*
Willpowery.RT	*WP_12.y*	*0.436*	*0.058*	*7.54*	*< .001*	*0.436*	*3.30*

Associations with Two Subscales of Willpower Belief Measure

Because the willpower belief questionnaire formed two subscales—the Resisting Temptations subscale and the Strenuous Mental Effort subscale—we repeated the analyses in the main manuscript using each of the two subscales separately to see whether the results are specific to one particular type of willpower belief.

Support

The two subscales were somewhat associated with the receipt and provision of social support to different extents, although all relationships were negative (see Figure S5).

The Strenuous Mental Effort subscale (6 items) was less consistently associated with the receipt or provision of support (compared to the overall scale or the Resisting Temptations

subscale). According to the perspective of the support recipient only, people with more limited strenuous mental effort beliefs were less likely to provide support (both instrumental and emotional). Additionally, only according to the support provider, those with limited theories of strenuous mental effort were less likely to receive instrumental social support (no significant association with emotional support).

The Resisting Temptations subscale (four items; other two items excluded due to low loadings) was consistently associated with lower provision of social support and lower receipt of social support (even more consistently associated than the overall 12-item scale). Participants who were better able to resist temptations were more likely to provide support to their partners (according to the perspective of both partners, both instrumental and emotional support) and were also more likely to receive support from their partners (according to the perspective of both partners, both instrumental and emotional support).

Figure S5

Summary of Eight Statistical Models Examining How Support was Predicted by Both Willpower Belief Subscales of Both Relationship Partners

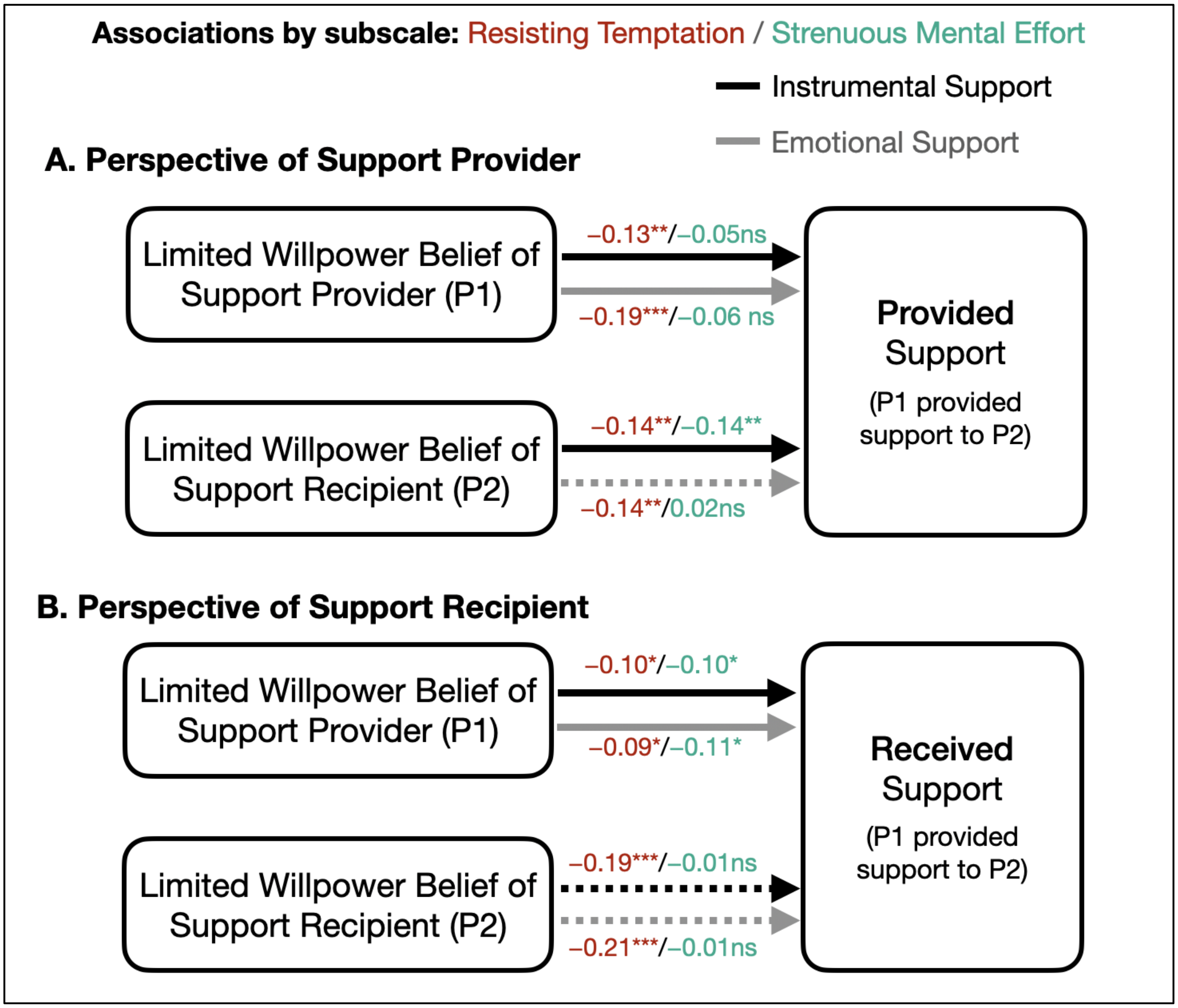

Note. The solid lines reflect cases where the overall willpower belief (across all 12 items) was significantly associated with support, whereas the dashed line reflect non-significant associations with the overall willpower believe scale (i.e., the solid or dashed lines are consistent with the lines as shown in Figure 2 of the main paper, to allow for easier comparison).

Relationship Satisfaction

As described in the main manuscript, daily relationship satisfaction was significantly predicted by one's own willpower belief (when measured with the entire willpower belief scale)

but not by one's partner willpower belief. In other words, there was a significant actor-effect of willpower belief on relationship satisfaction, but no partner-effect. This same pattern was observed for the Strenuous Mental Activity subscale, where the actor-effect was significant ($b = -0.04$, $SE = 0.01$, $t(708) = -2.67$, $p = .008$) but the partner-effect was not ($b = 0.02$, $SE = 0.01$, $t(709) = 1.27$, $p = .204$). However, when looking at the Resisting Temptation subscale, there was both a significant actor-effect ($b = -0.04$, $SE = 0.01$, $t(711) = -3.07$, $p = .002$) and a significant negative partner-effect ($b = -0.04$, $SE = 0.01$, $t(712) = -2.64$, $p = .009$).

Based on these data, beliefs about one's ability to resist temptation may be more strongly affected by one's relationship satisfaction and by one's receipt of and provision of support. People who are in more supportive, higher quality relationships tend to report higher beliefs in their ability to resist temptations.

Similarity Effects

The results for the similarity effects for each subscale examined separately all generally followed the same patterns as the overall willpower belief scale; similarity of neither the strenuous mental activity beliefs nor resisting temptation beliefs was significantly associated with relationship satisfaction ($ps > .25$).

Like the overall scale, partners' strenuous mental activity beliefs correlated weakly with one another ($r(580) = 0.09$, $p = .025$). Resisting temptation beliefs had a similarly weak correlation coefficient, although it did not reach statistical significance ($r(596) = 0.07$, $p = .081$).

References

Krohne, H. W., Egloff, B., Kohlmann, C. W., & Tausch, A. (1996). Investigations with a German version of the positive and negative affect schedule (PANAS). *Diagnostica, 42*(2), 139-156

Selig, J. P., & Preacher, K. J. (2008, June). *Monte Carlo method for assessing mediation: An interactive tool for creating confidence intervals for indirect effects* [Computer software]. Available from http://quantpsy.org/.

Watson, D., Clark, L. & Tellegen, A. (1988). Development and validation of brief measures of positive and negative affect: The PANAS scales. *Journal of Personality and Social Psychology*, *54*, 1063-1070.
